# Crystal settling and convection in the Shiant Isles Main Sill

**DOI:** 10.1007/s00410-016-1325-x

**Published:** 2017-01-19

**Authors:** Marian B. Holness, Robert Farr, Jerome A. Neufeld

**Affiliations:** 1 Department of Earth Sciences, University of Cambridge, Downing Street, Cambridge, CB2 3EQ UK; 2 Unilever Research and Development, Colworth Science Park, Bedford, MK44 1LQ UK; 3 BP Institute for Multiphase Flow, University of Cambridge, Madingley Road, Cambridge, CB3 0EZ UK; 4 Department of Applied Mathematics and Theoretical Physics, Centre for Mathematical Sciences, University of Cambridge, Wilberforce Road, Cambridge, CB3 0WA UK

**Keywords:** Convection, Crystallisation, Microstructure, Grain size, Composite intrusion

## Abstract

The 168 m-thick Shiant Isles Main Sill is a composite body, dominated by an early, 24 m-thick, picrite sill formed by the intrusion of a highly olivine-phyric magma, and a later 135 m-thick intrusion of olivine-phyric magma that split the earlier picrite into a 22 m-thick lower part and a 2 m-thick upper part, forming the picrodolerite/crinanite unit (PCU). The high crystal load in the early picrite prevented effective settling of the olivine crystals, which retain their initial stratigraphic distribution. In contrast, the position of the most evolved rocks of the PCU at a level ~80% of its total height point to significant accumulation of crystals on the floor, as evident by the high olivine mode at the base of the PCU. Crystal accumulation on the PCU floor occurred in two stages. During the first, most of the crystal load settled to the floor to form a modally and size-sorted accumulation dominated by olivine, leaving only the very smallest olivine grains still in suspension. The second stage is recorded by the coarsening-upwards of individual olivine grains in the picrodolerite, and their amalgamation into clusters which become both larger and better sintered with increasing stratigraphic height. Large clusters of olivine are present at the roof, forming a foreshortened mirror image of the coarsening-upwards component of the floor accumulation. The coarsening-upwards sequence records the growth of olivine crystals while in suspension in a convecting magma, and their aggregation into clusters, followed by settling over a prolonged period (with limited trapping at the roof). As olivine was progressively lost from the convecting magma, crystal accumulation on the (contemporaneous) floor of the PCU was increasingly dominated by plagioclase, most likely forming clusters and aggregates with augite and olivine, both of which form large poikilitic grains in the crinanite. While the PCU is unusual in being underlain by an earlier, still hot, intrusion that would have enhanced any driving force for convection, we conclude from comparison with microstructures in other sills that convection is likely in tabular bodies >100 m thickness.

## Introduction

The processes involved in creating layered intrusions have recently been the subject of some dispute (e.g. Latypov 2009; Latypov et al. 2015; Marsh 2015). Critical to many of the arguments is the fluid dynamical behaviour of crystal-bearing magmas. While some workers argue that magma bodies undergo vigorous convection (Sparks 1990; Huppert and Turner 1991), others have argued that convection is absent (Gibb and Henderson 1992) or limited to an initial super-heated stage of the intrusion’s history (Marsh 1989, 1991). The disagreement could be resolved if consensus could be reached over the interpretation of features preserved in exposed examples of fully solidified intrusions. Evidence used to argue for convection includes: the preservation of depositional and erosional structures in the floor cumulates of the Skaergaard intrusion, indicative of significant flow of magma across the floor (Irvine et al. 1998); the development of identical mineral assemblages, comprising minerals with the same composition on the roof, walls and floor of individual km-scale chambers, implying effectively mixed magma on the intrusion-wide scale (e.g. Salmonsen and Tegner 2013); and the wholesale movement of solids from the roof to the floor of stationary, thick, lava flows, implying a corresponding upwards movement of displaced liquid (Philpotts and Dickson 2000; this is known as two-phase convection). Observations used to argue for static, non-convecting magma mainly concern the spatial distribution of phenocrysts: D-shaped distributions in sills can be understood as the result of flow concentration of phenocrysts in the centre of the flow, with no subsequent re-arrangement by convection or settling (Gibb and Henderson 1992). An S-shaped distribution is generally interpreted as a consequence of gravitational settling (e.g. Gibb and Henderson 1996) although these distributions have not been investigated to test the extent to which settling may have been slowed by convection.

In this contribution, we report the results of a microstructural investigation of the Shiant Isles Main Sill. The distribution of olivine in the main intrusion of this composite body has been proposed as evidence of crystal settling from an essentially static olivine-phyric magma (Gibb and Henderson 1996). We present microstructural data, together with a simplified treatment of settling of a polydisperse suspension from a convecting magma that support an early period immediately after intrusion during which most of the crystal cargo settled to the floor, followed by a period during which convection was sufficiently vigorous to have kept mm-scale olivine grains and clusters in suspension for protracted periods.

## The Shiant Isles Main Sill: geological setting

**Fig. 1:**
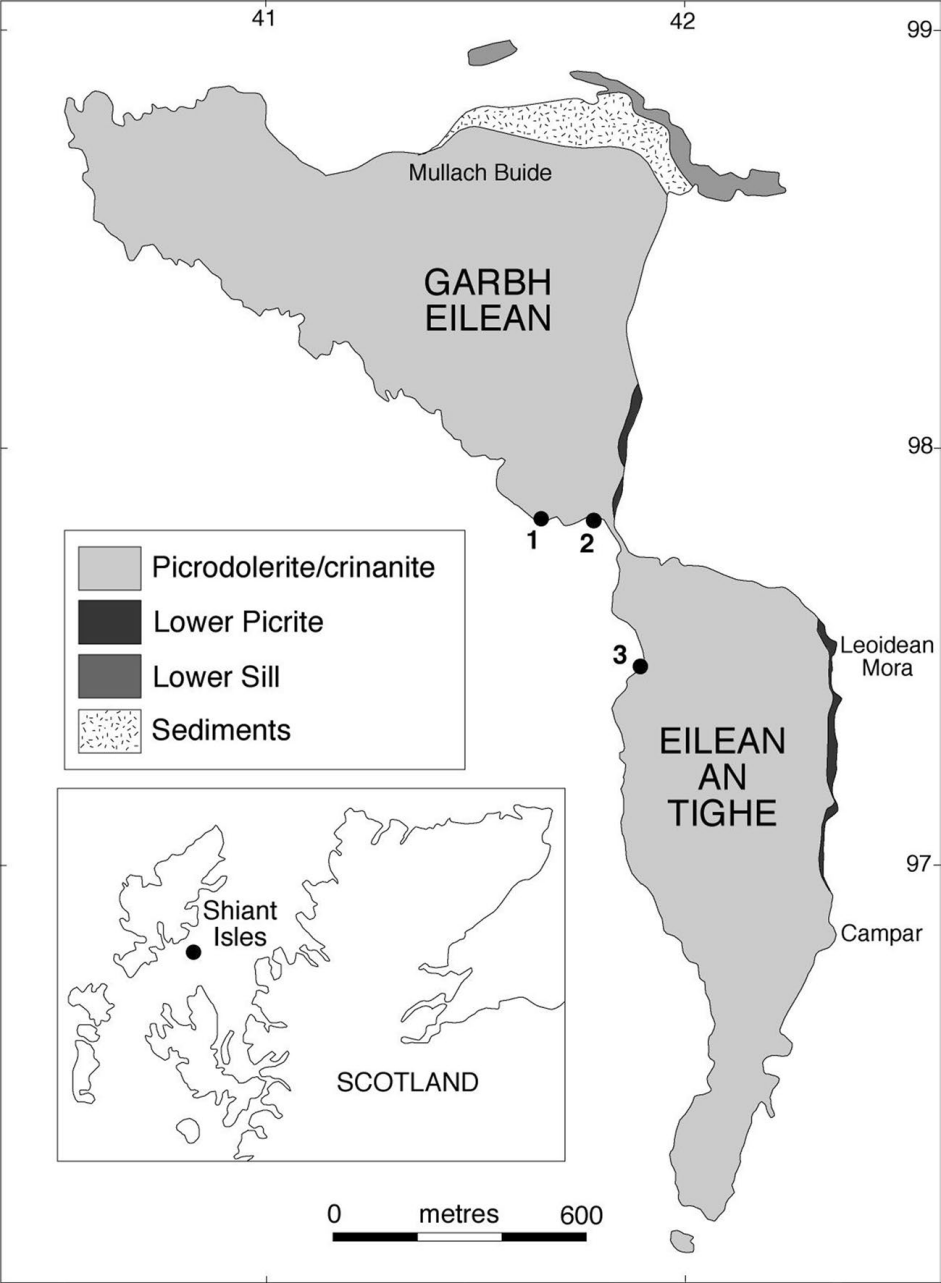
Simplified geological map of the Shiant Isles, after Gibb and Henderson (2006). The Lower Sill is not of concern here, while the two components of the Main Sill described in this contribution are the picrodolerite/crinanite unit (PCU) and the Lower Picrite. The numbered dots are the sites of the three drill cores with which the composite stratigraphy was established (Gibb and Henderson 1996)

Four Tertiary alkaline basalt sills are exposed on the Shiant Isles (Fig. 1), intruded into Jurassic sediments (Gibb and Henderson 1984). These sills are thought to comprise part of a larger sill complex that extends over much of the Little Minch and the Sea of the Hebrides, formed by repeated and often multiple intrusions of picritic and crinanitic magmas (Gibb and Gibson 1989).

The thickest of the sills exposed on the Shiant Isles, known as the Shiant Isles Main Sill, is a composite body (Drever and Johnston 1959; Gibb and Henderson 1996, 1989). The earliest intrusion is a 2 m-thick olivine teschenite (containing phenocrysts of olivine and plagioclase). This was followed by the intrusion of a 24 m-thick picrite magma containing abundant olivine phenocrysts. The third intrusion is 135 m thick where sampled by the drill cores (Fig. 1) and comprises the bulk of the sill. It was formed from an olivine-phyric magma containing ~ 10 vol.% olivine phenocrysts, together with 1–2 wt% Cr-spinel and a small amount of plagioclase (Gibb and Henderson 2006), that was emplaced high in the picrite unit before the host rock was completely solidified, splitting the picrite into upper (~2 m thick) and lower (~22 m thick) leaves. Gibb and Henderson (1992, 1996, 2006, 2014) suggest that the olivine phenocrysts settled to the floor of the 135 m-thick unit to form a picrodolerite, leaving an essentially aphyric magma that crystallised to form the remainder (crinanite) of this unit. A final injection of olivine-phyric magma, at the original upper contact between the picrite and the picrodolerite/crinanite unit (PCU), formed an ~4 m-thick intrusion of granular olivine picrodolerite. This last stage is thought to have eroded at least part of the original topmost parts of the crinanite (Henderson et al. 2000). The spectacular columnar jointing in the composite sill continues across the internal contacts, demonstrating that it behaved as a single unit during most of its cooling history and therefore that the four intrusive pulses were separated by short time intervals (Gibb and Gibson 1989), consistent with isotopic evidence for some mixing across the contacts between the different components (Foland et al. 2000).

The model of a composite sill was recently questioned by Latypov and Chistyakova (2009), who argued that the entire 168 m-thick sill formed by closed-system fractional crystallisation of a single pulse of olivine-saturated magma. Latypov later partially retracted this idea, suggesting instead that the observed mineralogical variations are the result of prolonged filling of the sill by magma of an increasingly primitive composition, followed by closed-system fractional crystallisation (Latypov 2014).

**Fig. 2:**
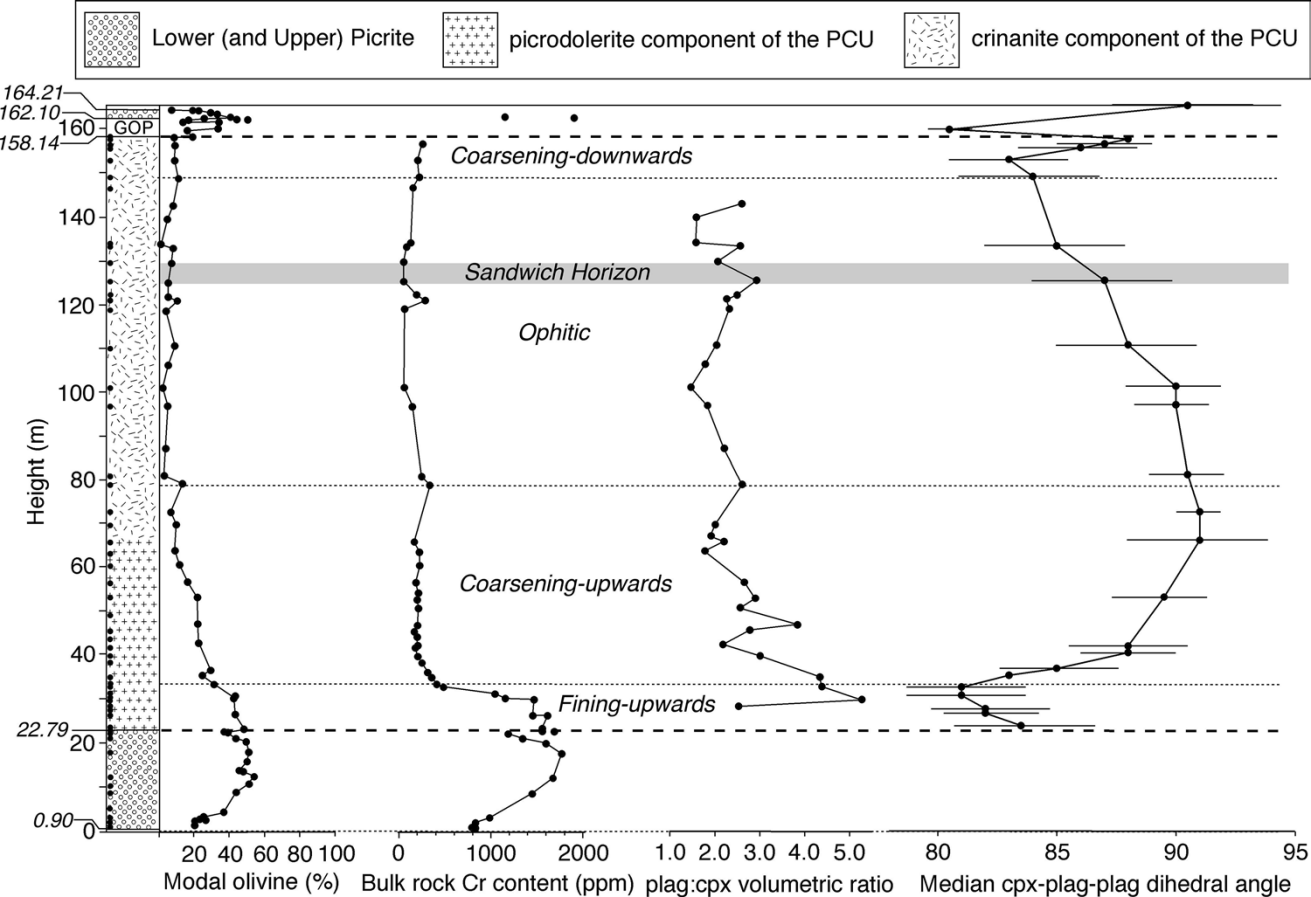
The stratigraphy of the Shiant Isles Main Sill (simplified from Gibb and Henderson (1996), omitting the bands of pegmatite at the top of the PCU and which were not examined during this study. GOP shows the stratigraphic distribution of the granular olivine picrodolerite (see text for details)), together with the stratigraphic variation of olivine mode (from Henderson et al. 2000), bulk rock Cr content in Hole 1 (from Fig. 8 of Gibb and Henderson (2006). Location shown in Fig. 1), augite-plagioclase-plagioclase dihedral angle, and the volumetric ratio of plagioclase to clinopyroxene (data from Gibb and Henderson 1996). The stratigraphic heights of the samples examined for the present study are shown by the black dots on the *left side* of the stratigraphic column. The * horizontal dashed lines* show the margins of the picrodolerite/crinanite unit, while the * horizontal dotted lines* show the boundaries between the four different subdivisions of the PCU on the basis of olivine morphology. The boundary between the fining-upwards and coarsening-upwards subdivisions is gradational (Fig. 6). The * grey band* shows the stratigraphic position of the most evolved rocks (from Gibb and Henderson 2006) which is the Sandwich Horizon where the roof and floor sequences meet

We focus here on the two components that comprise the bulk of the composite sill: the lower, ~22 m thick, part of the 24 m-thick picrite sill (the second batch of magma to be intruded), termed the Lower Picrite, and the immediately overlying 135 m-thick picrodolerite/crinanite unit (or PCU)—the third and largest magma batch. We used a sub-set of 64 samples from the suite collected by Fergus Gibb and Michael Henderson, now held by the British Geological Survey. Gibb and Henderson (1996) developed a composite stratigraphy to link three separate drill cores (the locations of which are shown in Fig. 1) with surface samples: we use this stratigraphy here. We report microstructural and modal data for 53 samples (the stratigraphic locations of which are shown in Fig. 2) while the remaining samples, from the upper and lower chill zones, were used to make inferences about the incoming crystal load.

## Analytical methods

### Dihedral angle measurements

Dihedral angles at augite–plagioclase–plagioclase junctions were measured in 23 samples from the PCU using a 4-axis universal stage mounted on a J Swift monocular microscope, with a UM32 Leitz long- working-distance objective and a ×10 eyepiece. We report the median values of populations of up to 100 individual measurements of true 3-D dihedral angles in each sample. The 95% confidence intervals about the median were calculated according to the method of Stickels and Hücke (1964).

### Grain size measurements

Grain intersections for olivine were obtained from photomicrographs by drawing by hand around the outlines of olivine grains, taking care to distinguish between individual grains within polycrystalline olivine clusters by comparing the photomicrographs with observations using an optical microscope. The scanned line drawings were analysed using ImageJ to calculate the area of each intersected grain. These area values were converted to the equivalent diameter of a circular grain intersection (the Heywood diameter). The size of olivine clusters was determined in the same way. Although the shape of the clusters is highly irregular, these area values were also converted to their Heywood diameters.

A measure of the effect of overgrowth of olivine phenocrysts by crystallisation of the interstitial liquid in the crystal mush was made by comparing the actual perimeter of each grain intersection (as measured using ImageJ) with the circumference of a circle with the same area. For perfectly circular grain intersections the ratio of these two values is 1, whereas the ratio for more irregularly shaped grains (i.e. those for which there has been extensive post-accumulation overgrowth) is >1.

For simplicity, we report the average size of individual grains and clusters. While the true, 3D, distribution of olivine grain sizes can be obtained from stereological correction of the distribution of 2D grain intersections (e.g. Russ 1986), the approximate value of the (true 3D) grain diameter for a monodisperse population of spherical grains can be obtained by multiplying the mean diameter of the grain intersections by a factor of 1.274 (e.g. Cashman and Marsh 1988; Kong et al. 2005). However, this simple relationship does not hold for polydisperse populations.

For polydisperse populations, there are different ways to quantify the average (3D) diameter, and the choice of which to use depends on the problem under consideration (Farr et al. 2016). The volume-weighted mean diameter, D_4,3_ (see “Appendix”) represents the size around which most of the mass of the particles lies and is therefore a good measure of particle size for problems relating to composition. The area-weighted mean diameter, D_3,2_, is the mean obtained when spheres are chosen with a probability proportional to their surface area. The area-weighted mean diameter is most appropriate for problems involving surface area, such as the extent and rate of adsorption.

Olivine grain size and shape information were collected for 28 samples from the Lower Picrite and PCU. The upper parts of the sill (the granular olivine picrodolerite, the olivine teschenite and the upper leaf of the early picrite) are generally too altered for detailed microstructural work. In the 18 PCU samples in which the olivine mode was sufficiently low to permit the distinction between different clusters of olivine grains, the Heywood diameters of both individual grains and clusters were obtained. In Table 1, we provide details of the grain size populations, including the volume and area-weighted average diameters, together with a measure of the spread of the grain size population, σ, and an estimate of the uncertainty on the mean diameter, *e* (see “Appendix” for definitions).

**Table 1:** Microstructural data for olivine in the Shiant Isles Main Sill (see “Appendix” for definitions of the different measures of grain size)

			Individual olivine grains												Olivine clusters								
Sample	height (m)	vol.%	*n*_*g*_	Irreg	Σd	Σd^2^	Σd^3^	Σd^4^	D_4,3_	D_3,2_	D_3,1_	Corrected D_4,3_	σ	*e*	*n*_*c*_	Σd	Σd^2^	Σd^3^	Σd^4^	D_4,3_	D_3,2_	D_3,1_	Corrected D_4,3_
Lower picrite																							
SC744	2.28	*20.0*	*249*	1.15	71.11	25.32	11.12	5.93	0.50	0.42	0.39	0.44 ± 0.04	0.41	0.07	–	–	–	–	–	–	–	–	–
SC728	4.17	36.3	*385*	1.10	106.43	37.30	15.87	7.82	0.48	0.41	0.38	0.41 ± 0.03	0.39	0.05	–	–	–	–	–	–	–	–	–
SC681	8.70	43.6	*429*	1.09	116.90	41.50	18.33	9.62	0.50	0.42	0.38	0.44 ± 0.03	0.42	0.06	–	–	–	–	–	–	–	–	–
SC656	12.27	54.0	*622*	1.10	180.90	63.59	26.40	12.62	0.47	0.41	0.39	0.39 ± 0.02	0.36	0.04	–	–	–	–	–	–	–	–	–
SC598	17.91	51.2	*395*	1.12	119.74	45.19	20.69	11.15	0.52	0.44	0.41	0.44 ± 0.03	0.39	0.05	–	–	–	–	–	–	–	–	–
SC560	21.16	43.0	*364*	1.11	130.01	57.98	30.63	18.28	0.60	0.53	0.49	0.50 ± 0.03	0.36	0.05	–	–	–	–	–	–	–	–	–
SC539	22.43	36.3	*335*	1.09	129.92	61.79	35.23	23.54	0.65	0.56	0.53	0.54 ± 0.03	0.38	0.05	–	–	–	–	–	–	–	–	–
Picrodolerite/crinanite unit (PCU)																							
SC533	22.92	48.5	*431*	1.07	105.63	41.72	23.60	17.27	0.64	0.47	0.38	0.66 ± 0.08	0.57	0.12	–	–	–	–	–	–	–	–	–
SC518	23.64	48.6	*601*	1.09	144.46	54.06	29.36	21.26	0.61	0.44	0.37	0.64 ± 0.07	0.58	0.11	–	–	–	–	–	–	–	–	–
SC492	26.42	43.3	*617*	1.16	134.95	43.41	23.28	14.73	0.61	0.38	0.32	0.71 ± 0.11	0.69	0.19	–	–	–	–	–	–	–	–	–
SC459	29.72	*40.0*	*517*	1.07	98.05	30.37	14.13	8.84	0.53	0.36	0.30	0.57 ± 0.07	0.61	0.14	*256*	63.60	30.67	22.73	21.55	0.84	0.57	0.42	0.91 ± 0.19
S81/10	32.95	32.4	*519*	1.07	98.23	26.43	9.82	4.78	0.42	0.32	0.28	0.42 ± 0.04	0.53	0.09	*236*	57.58	23.10	13.51	9.90	0.66	0.47	0.38	0.69 ± 0.12
S81/12	35.08	24.5	*516*	1.14	101.22	29.24	12.33	7.14	0.48	0.34	0.29	0.50 ± 0.06	0.58	0.12	*228*	64.23	29.80	20.33	17.85	0.77	0.55	0.44	0.81 ± 0.15
S81/14	38.43	*23.0*	*455*	1.09	90.84	26.55	11.10	6.11	0.47	0.34	0.30	0.49 ± 0.06	0.56	0.12	*188*	55.06	26.02	17.01	13.49	0.74	0.56	0.46	0.74 ± 0.12
S81/15	39.95	*21.0*	*551*	1.16	114.02	34.39	15.42	9.70	0.51	0.36	0.31	0.54 ± 0.06	0.60	0.13	*202*	64.35	35.22	28.74	31.54	0.92	0.64	0.51	0.97 ± 0.20
S81/17	42.40	22.8	*609*	1.19	121.84	36.53	19.26	17.20	0.60	0.35	0.30	0.72 ± 0.14	0.72	0.23	*197*	64.50	37.45	37.18	54.26	1.12	0.68	0.53	1.29 ± 0.47
S81/19	45.57	16.9	*304*	1.14	81.32	30.17	15.63	11.17	0.59	0.44	0.39	0.59 ± 0.08	0.54	0.13	*91*	57.44	50.57	54.96	68.35	1.23	1.04	0.91	1.12 ± 0.14
SC253	49.28	*20.0*	*216*	1.19	62.55	23.67	11.44	6.82	0.55	0.45	0.41	0.50 ± 0.05	0.45	0.10	*64*	36.98	28.99	30.75	41.88	1.20	0.92	0.82	1.18 ± 0.28
S81/22	52.88	21.8	*171*	1.17	56.10	24.95	14.81	10.99	0.67	0.52	0.47	0.65 ± 0.09	0.50	0.14	*41*	25.58	21.60	22.88	28.39	1.20	0.99	0.89	1.10 ± 0.22
S81/24	56.57	16.1	*275*	1.17	82.10	35.57	20.62	15.17	0.66	0.51	0.44	0.63 ± 0.07	0.50	0.11	*92*	60.89	56.47	67.14	96.26	1.35	1.09	0.96	1.26 ± 0.19
S81/25	60.50	11.8	*376*	1.20	108.12	41.96	20.61	12.29	0.56	0.46	0.41	0.50 ± 0.04	0.44	0.07	*37*	34.02	42.21	59.70	91.20	1.60	1.46	1.31	1.36 ± 0.16
S81/26	63.64	8.8	*221*	1.29	90.59	51.05	36.95	32.72	0.82	0.66	0.59	0.75 ± 0.08	0.46	0.10	*57*	45.12	51.91	73.42	117.98	1.60	1.36	1.17	1.48 ± 0.23
S81/27	65.86	20.1	*247*	1.27	103.01	62.83	52.73	56.87	0.95	0.72	0.62	0.94 ± 0.13	0.53	0.13	*36*	37.60	63.26	134.82	329.63	2.41	1.98	1.62	2.22 ± 0.51
S81/29	69.68	10.0	*126*	1.50	63.65	44.97	39.26	39.08	0.99	0.83	0.73	0.87 ± 0.10	0.41	0.11	*33*	30.92	45.23	81.68	166.95	2.04	1.72	1.43	1.88 ± 0.40
S81/30	72.27	6.7	*219*	1.17	84.64	48.85	37.69	35.41	0.87	0.68	0.58	0.84 ± 0.11	0.50	0.12	*36*	34.57	48.78	87.28	182.93	2.03	1.66	1.43	1.94 ± 0.46
SC1160	148.71	11.7	*127*	–	60.52	52.90	66.19	99.30	1.42	1.03	0.79	1.46 ± 0.32	0.56	0.22	*56*	43.34	52.93	80.73	138.93	1.73	1.44	1.19	1.63 ± 0.27
SC1131	152.63	9.1	*454*	–	125.02	48.01	23.89	14.24	0.56	0.45	0.40	0.52 ± 0.04	0.47	0.07	*158*	74.65	48.46	39.02	36.58	0.91	0.76	0.68	0.87 ± 0.08
SC1081	156.16	9.2	*249*	–	79.58	37.14	22.25	15.66	0.68	0.55	0.47	0.62 ± 0.06	0.46	0.09	*144*	61.87	37.13	27.67	24.13	0.84	0.71	0.62	0.81 ± 0.08
SC1072	157.29	*8.9*	*154*	–	59.61	27.28	13.95	7.73	0.58	0.54	0.52	0.44 ± 0.03	0.27	0.05	*105*	49.50	27.37	17.06	11.63	0.71	0.65	0.63	0.65 ± 0.04
SC1062	158.11	*11.1*	*190*	–	60.43	26.37	14.13	8.70	0.61	0.51	0.46	0.53 ± 0.05	0.41	0.08	*156*	55.98	26.27	14.71	9.29	0.63	0.55	0.50	0.58 ± 0.04

Height gives the stratigraphic height in the composite profile of Gibb and Henderson (1996). The number of individual grains and the number of olivine clusters analysed in each sample is given by *n*_*g*_ and *n*_*c*_. “Irreg” gives the irregularity of the grains (see text for explanation). The olivine mode (vol.%) is taken from Gibb and Henderson (1996) and Henderson et al. (2000): those values in italics were either interpolated from available data or measured from thin sections as part of this study

To assess the original size of the olivine grains, the sizes observed in the fully solidified rock were corrected to account for post-accumulation overgrowth of the original phenocrysts during solidification of the interstitial liquid. For this correction (for details, see “Appendix”), we assume that olivine crystallising from the interstitial liquid will overgrow pre-existing grains rather than nucleate new grains, and that overgrowth occurred only from the interstitial liquid in the immediate surroundings (i.e. on the thin-section scale). The composition of the interstitial liquid is taken to be that of the liquid PicrPL of Gibb and Henderson (2006) for the Lower Picrite, and the liquid PdolPL1 (Gibb and Henderson 2006) for the lower 10 m of the picrodolerite in the PCU (the lowermost 5 PCU samples examined here). For the remainder of the PCU, we assume the composition of the interstitial liquid changed linearly through the remaining picrodolerite from PdolPL1 to that of the liquid which solidified to form the crinanite. For the Lower Picrite, we also assume that olivine is the only accumulating phase (i.e. that all other phases grew solely from the interstitial liquid). We argue later that olivine accumulated together with plagioclase in the lower part of the PCU. Accordingly, we assumed that the volume fraction of liquid in the accumulated pile remained constant at 55 vol.% in the lower 10 m of the picrodolerite, rising to 60 vol.% in the upper part of the picrodolerite in which plagioclase became dominant (see later for arguments justifying this).

Our correction for overgrowth introduces some (unknown) degree of bias because many olivine grains in the Shiant Isles Main Sill form clusters. Those grains in the centres of the clusters were unlikely to have been in contact with interstitial liquid and therefore did not grow post-accumulation. If clustering occurred before overgrowth, all the overgrowth will therefore occur on the outermost grains while those in the centre of clusters will remain the same size. A bias might therefore be introduced if the clustering were not fully random, with large and small grains distributed irregularly within each cluster. We have not considered the magnitude of this effect in the present contribution.

## The Shiant Main Sill: petrography

The composite sill is dominated by plagioclase, augite, olivine and oxides. Mineral compositional data are presented by Gibb and Henderson (1996, 2006), and modal data are presented by Gibb and Henderson (1996): the published olivine modes, bulk rock Cr contents, and plagioclase:augite volumetric ratios are reproduced in Fig. 2. The most evolved bulk compositions in the PCU occur between 125 and 130 m stratigraphic height (Gibb and Henderson 2006) with the position marked in Fig. 2 as the Sandwich Horizon. Below we summarise microstructural features of interest to the present study: much of this repeats the full petrographic descriptions of Gibb and Henderson (1996).

### The chill zone

Generally, the later intrusions of composite bodies are emplaced within a pre-existing, partially solidified magma body. The chill zone against the country rocks should therefore only be formed from the first batch of magma. This is certainly the case for the lower boundary of the sill, for which picrite magma is chilled against the country rock (Gibb and Henderson 1984, 1989), and is locally the case for the upper contact (as shown in Fig. 7 of Gibb and Henderson 2006). However, there are localised regions of relatively coarse-grained crinanite at the upper contact, suggestive of transgressive intrusion into an already hot country rock (e.g. Gibb and Henderson 1989; Gibb, personal communication, 2015).

**Fig. 3:**
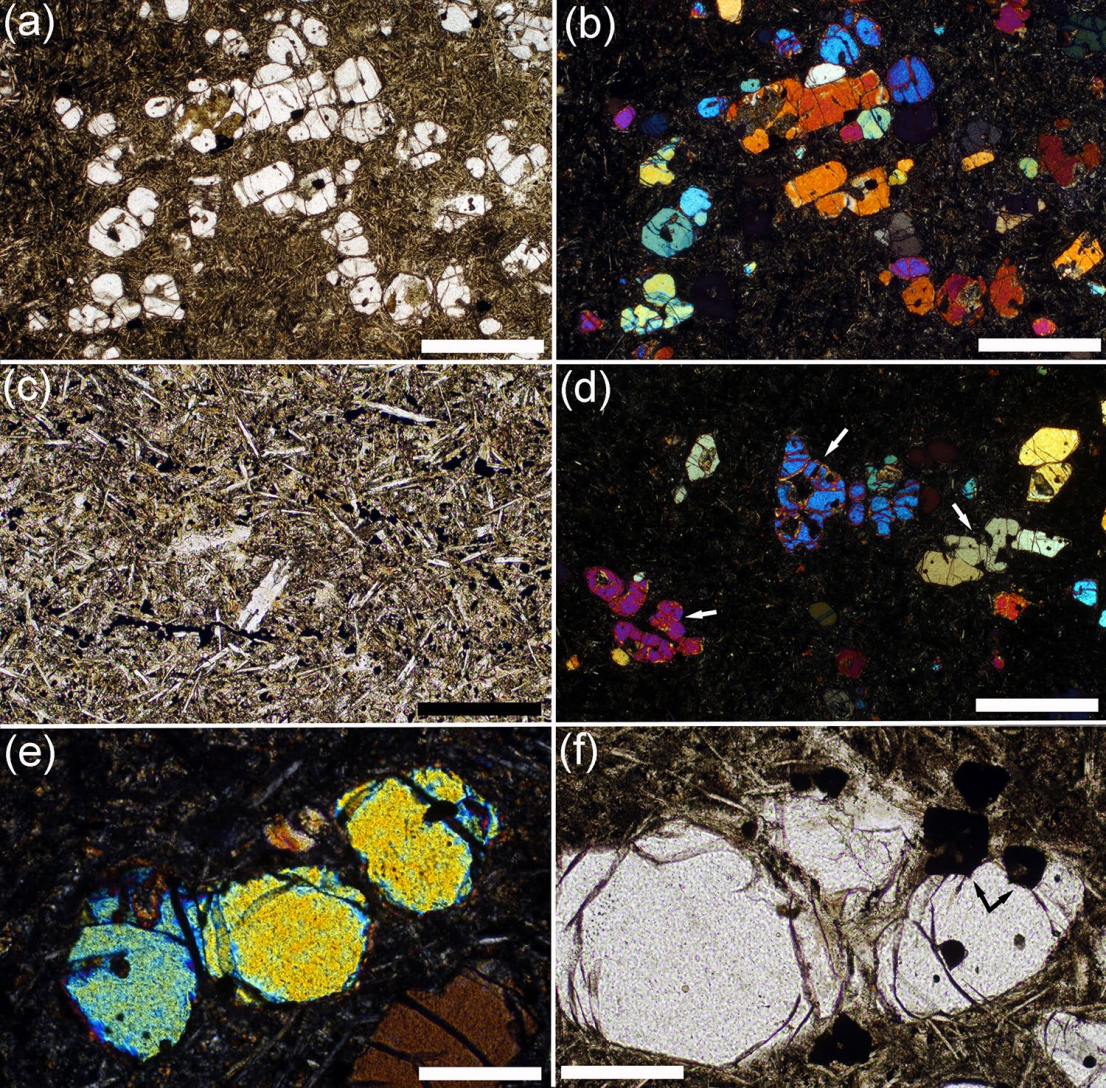
Photomicrographs of preserved chill zones of the Shiant Isles Main sill. **a** Olivine phenocrysts, together with small rounded grains of chromite, set in a fine-grained groundmass. Sample S82/41. Plane polarised light. *Scale bar* is 1 mm long. **b** Same field of view as **a** but with crossed polars. Note the grain clustering. *Scale bar* is 1 mm long. **c** Sample SC986 showing the details of the groundmass. Note the randomly oriented elongate plagioclase laths, set in a granular matrix dominated by pyroxene. Granular oxide grains commonly form strings with a preferred orientation but these are most likely recrystallized along fractures during hydrothermal alteration. Plane polarised light. *Scale bar* is 200 µm long. **d** Sample S82/41, under crossed polars, showing the non-compact structure of many of the olivine phenocrysts. Examples are *arrowed*. Many of these grains have a recognisably orthorhombic symmetry and therefore are likely to be partially annealed dendrites. Note that some clusters are formed of grains with very little crystallographic misorientation. *Scale bar* is 1 mm long. **e** Small cluster of rounded olivine grains in sample S82/41, joined by small areas of grain boundary. Note the similarity of birefringence colours: these three grains also have very similar extinction positions indicating low degrees of crystallographic misorientation. Crossed polars. *Scale bar* is 200 µm long. **f** Cluster of three olivine grains in sample S82/41, showing the close association with chrome spinel. The *arrowed* spinel grains contain irregular internal pockets filled with groundmass, indicative of dendritic growth. Plane polarised light. *Scale bar* is 200 µm long

Because both the picrite and the picrodolerite/crinanite units were formed of olivine-phyric magmas that are likely to have been related, the crystal load of both may have been similar. We therefore investigated two chilled picrite samples. The first, sample SC986, was collected from the top contact where intersected by Hole 3 (Gibb and Henderson 1984), while the second, S82/41, is from the lower contact on Garbh Eilean.

The picrite chill contains abundant olivine phenocrysts in a fine-grained groundmass (Fig. 3a, b). The groundmass contains randomly oriented swallowtail plagioclase set in granular pyroxene, together with oxides that locally form elongated clusters and chains with several preferred orientations (Fig. 3c). The multiplicity of preferred orientations suggests redistribution of oxide into fractures during later hydrothermal alteration.

**Fig. 4:**
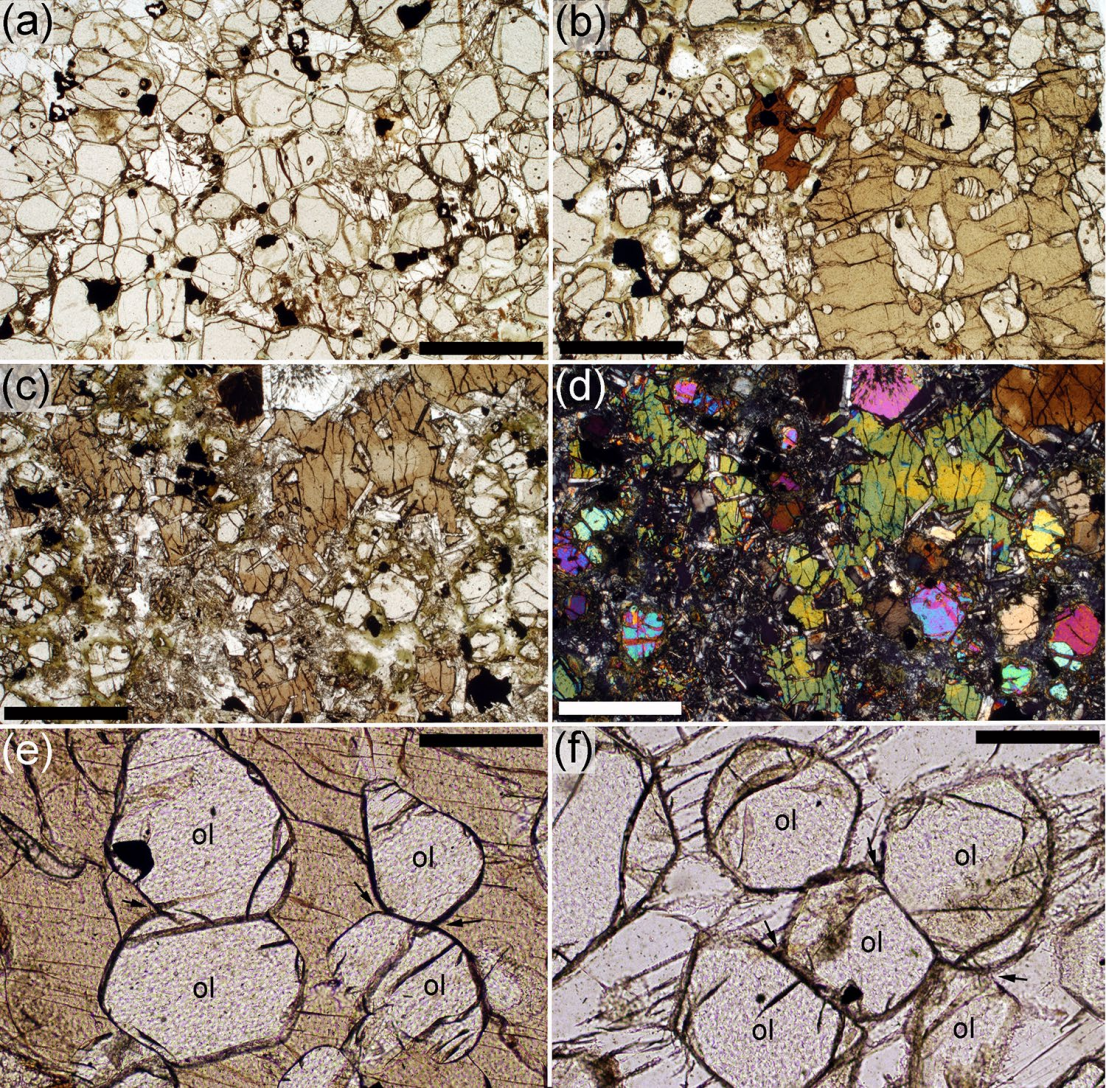
Photomicrographs of the Lower Picrite. **a** Sample SC598, from 17.91 m stratigraphic height. Note the high olivine mode and the generally uniform olivine grain size, together with the scattered abundance of Cr-spinel. Plane polarised light. *Scale bar* is 1 mm long. **b** Sample SC681, 8.7 m stratigraphic height. Note the *large, pale brown*, augite oikocryst in the right half of the image. The olivine grains enclosed by the augite are smaller, more irregularly shaped and less abundant than those surrounded by plagioclase. A grain of *dark brown* interstitial kaersutite is visible just above the centre of the image. Plane polarised light. *Scale bar* is 1 mm long. **c** Sample SC744, from 2.28 m stratigraphic height. Note the irregularly coloured brown oikocryst of augite, indicative of compositional zoning. Plane polarised light. *Scale bar* is 1 mm long. **d** Sample SC744, showing same field of view as **c** under crossed polars. *Scale bar* is 1 mm long. **e** Sample SC598, from 17.91 m stratigraphic height, showing the rounded morphology of olivine grains included in augite oikocrysts. Note the well-defined grain boundaries, smooth curvature at olivine–olivine–augite grain junctions and the low dihedral angles indicative of solid–solid–liquid textural equilibration (examples are *arrowed*—assuming that augite is pseudomorping the liquid phase). Plane polarised light. *Scale bar* is 200 µm long. **f** Sample SC598, from 17.91 m stratigraphic height, showing a cluster of five rounded olivine grains surrounded by interstitial plagioclase. The smooth grain boundaries and low olivine–olivine–plagioclase dihedral angles are suggestive of solid–solid–liquid textural equilibration of the olivine clusters (examples are arrowed—assuming plagioclase is pseudomorphing the liquid phase). Plane polarised light. *Scale bar* is 200 µm long

The olivine phenocrysts are bounded by growth facets joined by areas of rounded surface (Fig. 3d). Many have non-equant shapes indicative of a partially annealed dendrite (c.f. Welsch et al. 2013), while others are compact and equant. They form small clusters, with the grains joined by small areas of grain boundary. Some clusters are made of grains with very similar optical orientation, denoting similar crystallographic orientations and low-angle grain boundaries (Fig. 3e). Similar low mismatch angles in olivine clusters from Kilauea Iki are thought to denote synneusis in a liquid-rich environment (Schwindinger and Anderson 1989). Equant, commonly hollow, chrome spinel grains (also part of the crystal cargo) adhere to the olivine grains (Fig. 3f), though some appear fully enclosed by olivine.

### The lower picrite

**Fig. 5:**
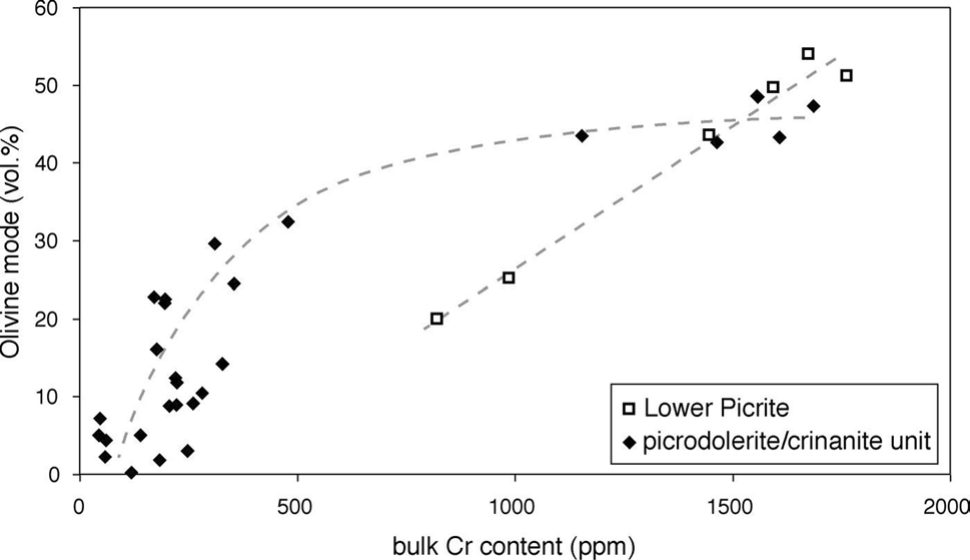
The relationship between the olivine mode [from Henderson et al. (2000)] and the bulk rock Cr content [from Gibb and Henderson (2006)] for the Lower Picrite and the picrodolerite/crinanite unit (PCU). The *dashed lines* showing the trends in the data are sketched by eye. Note that the olivine mode and bulk rock Cr content in the Lower Picrite and the basal (olivine-rich) region of the picrodolerite are indistinguishable, suggesting a similar load of olivine and Cr-spinel phenocrysts in the two incoming magma batches

The lower picrite contains abundant rounded olivine crystals, with interstitial plagioclase, augite, Fe-Ti oxides, and late-crystallising kaersutite (Fig. 4a, b). The interstitial augite is locally compositionally zoned (Fig. 4c, d). Cr-rich spinel is abundant, forming isolated euhedral grains enclosed by interstitial plagioclase and augite (Fig. 4a), and euhedral inclusions in the olivine (Fig. 4e). Chromite grains tend to be closely associated with olivine, in a similar manner to that observed in the chill (e.g. Fig. 3f). The bulk rock Cr concentration (an approximate proxy for the mode of Cr-spinel) is linearly correlated with the olivine mode (Fig. 5; data from Gibb and Henderson 2006).

**Fig. 6:**
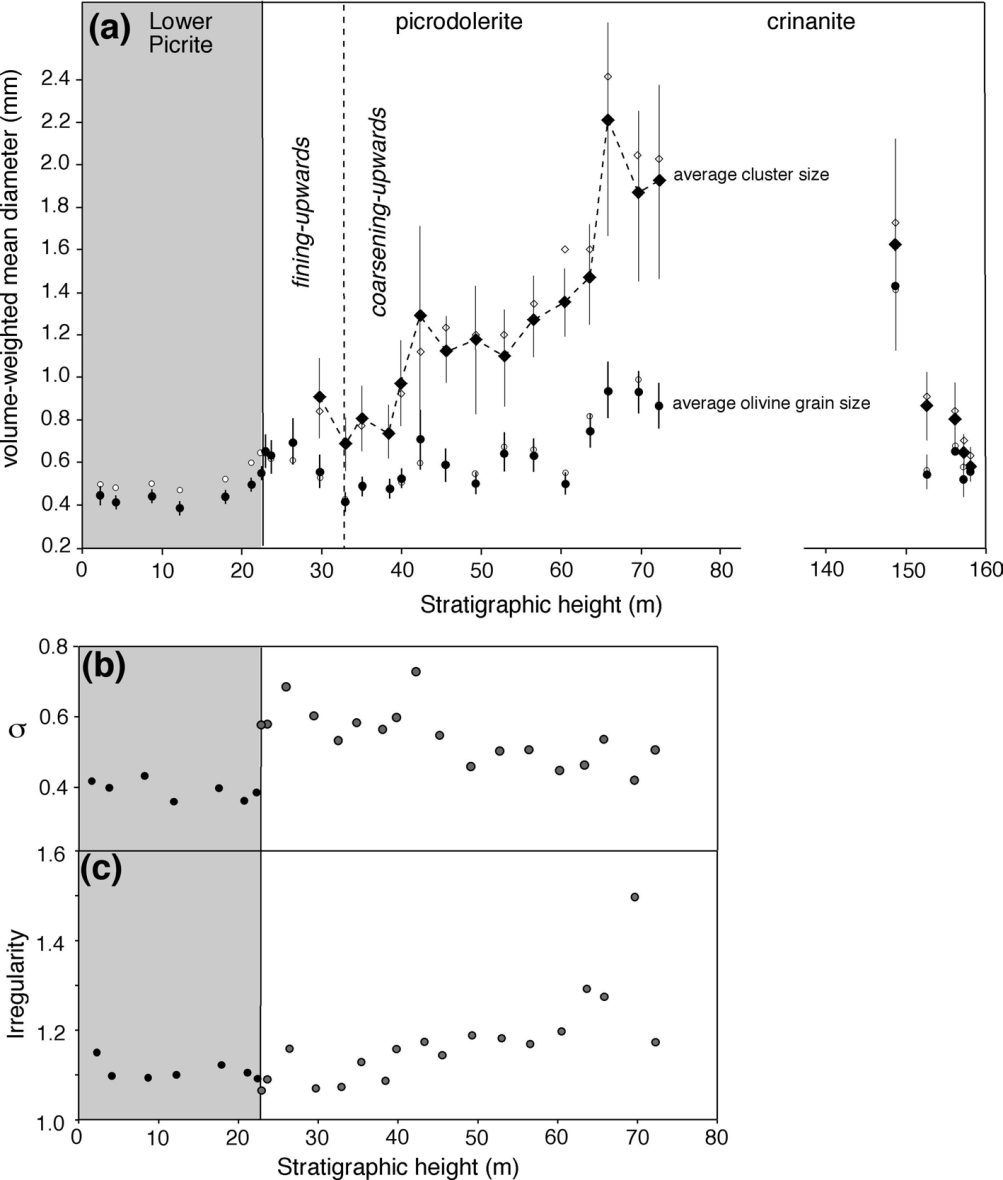
Size data for olivine in the Shiant Isles Main Sill. **a** The volume-weighted average diameters (D_4,3_) of individual olivine grains and olivine clusters in the Lower Picrite, and the picrodolerite/crinanite unit (PCU). Note the break in the *x* axis to enable ease of comparison of microstructures in the picrodolerite with those in the upper 10 m of the crinanite. The *black filled symbols* are the corrected averages, while the *open symbols* are the uncorrected averages. The uncertainties on the values of D_4,3_ are calculated according to the “Appendix” and provided in Table 1. The trend of the average diameter of the olivine clusters is shown by the dashed line to highlight the step-wise increase at ~40 m stratigraphic height. **b** The variation of the spread of the olivine grain diameters, as quantified by σ (see “Appendix” for details) in the Lower Picrite and picrodolerite component of the PCU. **c** The corresponding average irregularity of the shape of individual olivine grain intersections, where a value of 1.0 denotes a circular grain intersection and higher values denote increasing departure from a circle

The variation of olivine mode forms a near-symmetrical D-shaped profile across the Lower Picrite, reaching a maximum of 54 vol.% at 12.3 m stratigraphic height (Fig. 2; Gibb and Henderson 1996), attributed by Gibb and Henderson (1992, 1996) to the absence of post-intrusion settling or convective mixing. The olivine grains are well sorted, with a low value of σ (Table 1; Fig. 4a). The olivine is fine-grained and less-abundant where enclosed by augite oikocrysts, compared to where it is surrounded by plagioclase. Furthermore, the shapes of olivine grains included in augite oikocrysts are commonly irregular, perhaps indicative of partial dissolution (Fig. 4b).

The compilation of olivine grain size data in the Lower Picrite given in Table 1 is only for olivine grains outside augite oikocrysts. We corrected the average grain sizes assuming that olivine is the only accumulating phase and that the carrier liquid in the picrite has a composition close to the 5 kbar olivine–augite–plagioclase cotectic (liquid PicrPL of Gibb and Henderson (2006). Such a liquid crystallises ~10 vol.% olivine. The original olivine mode, ϕ_sed_, can be determined using *X* (vol%) = ϕ_sed_ + 0.1(100 − ϕ_sed_), where *X* is the actual olivine mode in the rock (i.e. the original mode plus the overgrowth).

The corrected average olivine grain sizes (reported as the volume-weighted mean diameter, D_4,3_) in the Lower Picrite are shown in Fig. 6a as a function of stratigraphic height. The average grain size is generally constant with height, with a smooth increase in the uppermost few metres (this holds true regardless of the choice of the measure of the average diameter). The spread, σ, of the grain size population (defined in the “Appendix”) is approximately constant with height (Fig. 6b). The average value of the ratio between the actual perimeter of each grain intersection and the perimeter of a circle with its Heywood diameter is in the range 1.09–1.15, denoting a close approximation to spherical grains (Fig. 6c).

**Fig. 7:**
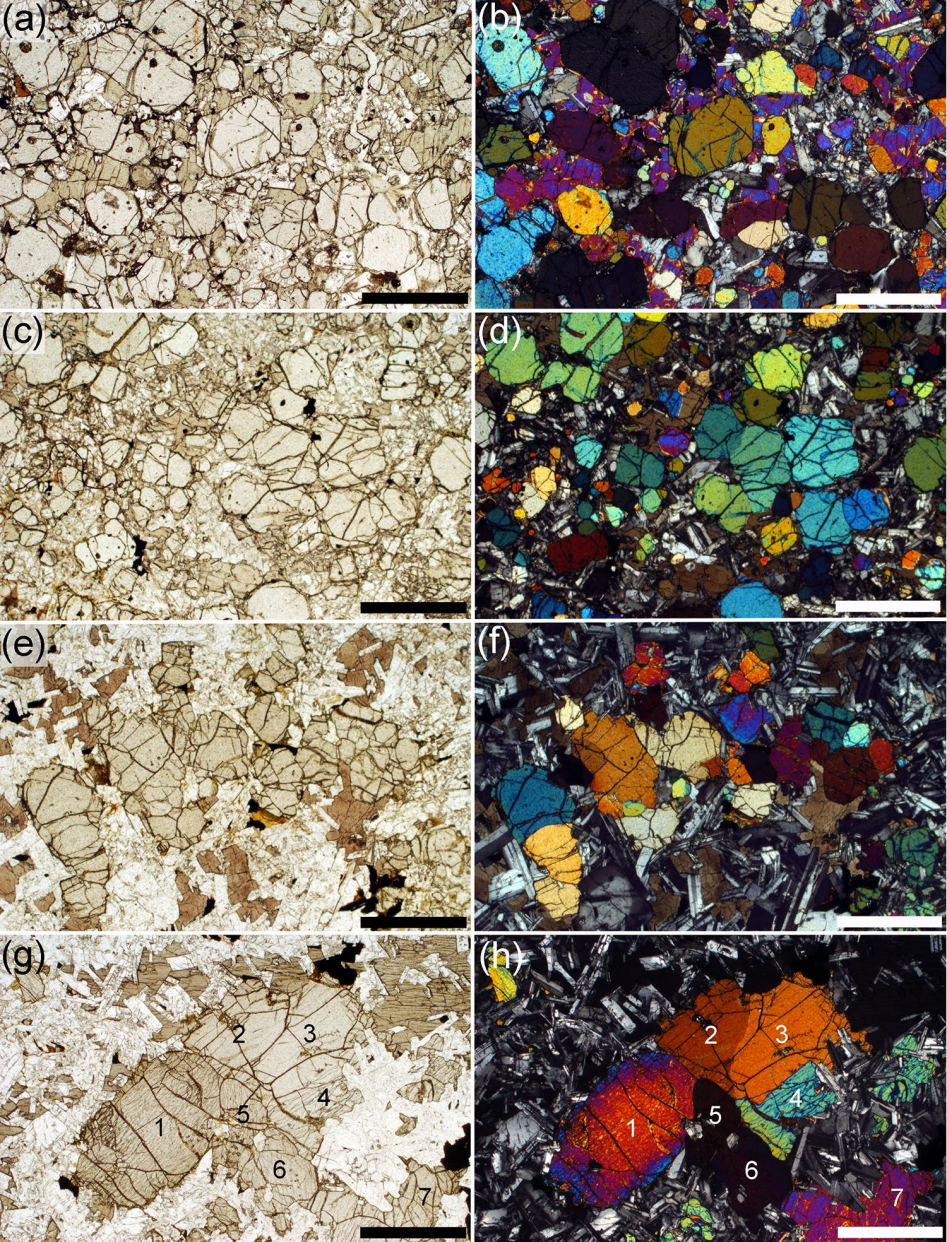
Photomicrographs of microstructures in the picrodolerite, with pairs of images photographed with plane polarised light and under crossed polars, showing the progressive change in olivine morphology. **a, b** Sample SC533, from 22.92 m stratigraphic height. Note the wide range of grain sizes and that the olivine grains commonly form loose clusters. Augite forms a single oikocryst in these two images. *Scale bar* is 200 µm long. **c, d** Sample S81/8, from 30.51 m stratigraphic height. In comparison with **a** and **b**, this sample contains very few apparently isolated grains, suggesting that most olivine forms loose clusters. *Scale bar* is 200 µm long. **e, f** Sample S81/24, from 56.57 m stratigraphic height. All olivine is found in clusters that are well-sintered with large areas of olivine–olivine grain boundary, no interstitial material and 120° triple junctions. *Scale bar* is 1 mm long. **g, h** S81/27, from 65.87 m stratigraphic height. Olivine is coarse-grained and forms well-sintered clusters with no interstitial material and 120° triple junctions. Note the prominent compositional zoning in the grains labelled 1 and 7, in comparison with grains 2, 3 and 4, which are enclosed by an augite oikocryst. *Scale bar* is 1 mm long

Although most olivine–olivine grain boundaries have been slightly serpentinised, they are generally smooth (Fig. 4e, f). The low olivine–olivine–augite (Fig. 4e) and olivine–olivine–plagioclase (Fig. 4f) dihedral angles and the smoothly curved interfaces between olivine and the adjacent plagioclase or augite are suggestive of some degree of textural equilibration with liquid before crystallisation was complete. The high olivine mode makes it difficult to determine whether the olivine grains originally formed very loose chains, small clusters, or settled as individual grains. None of the olivine grains has undulose extinction or contains sub-grains.

### The picrodolerite/crinanite unit (PCU)

**Fig. 8:**
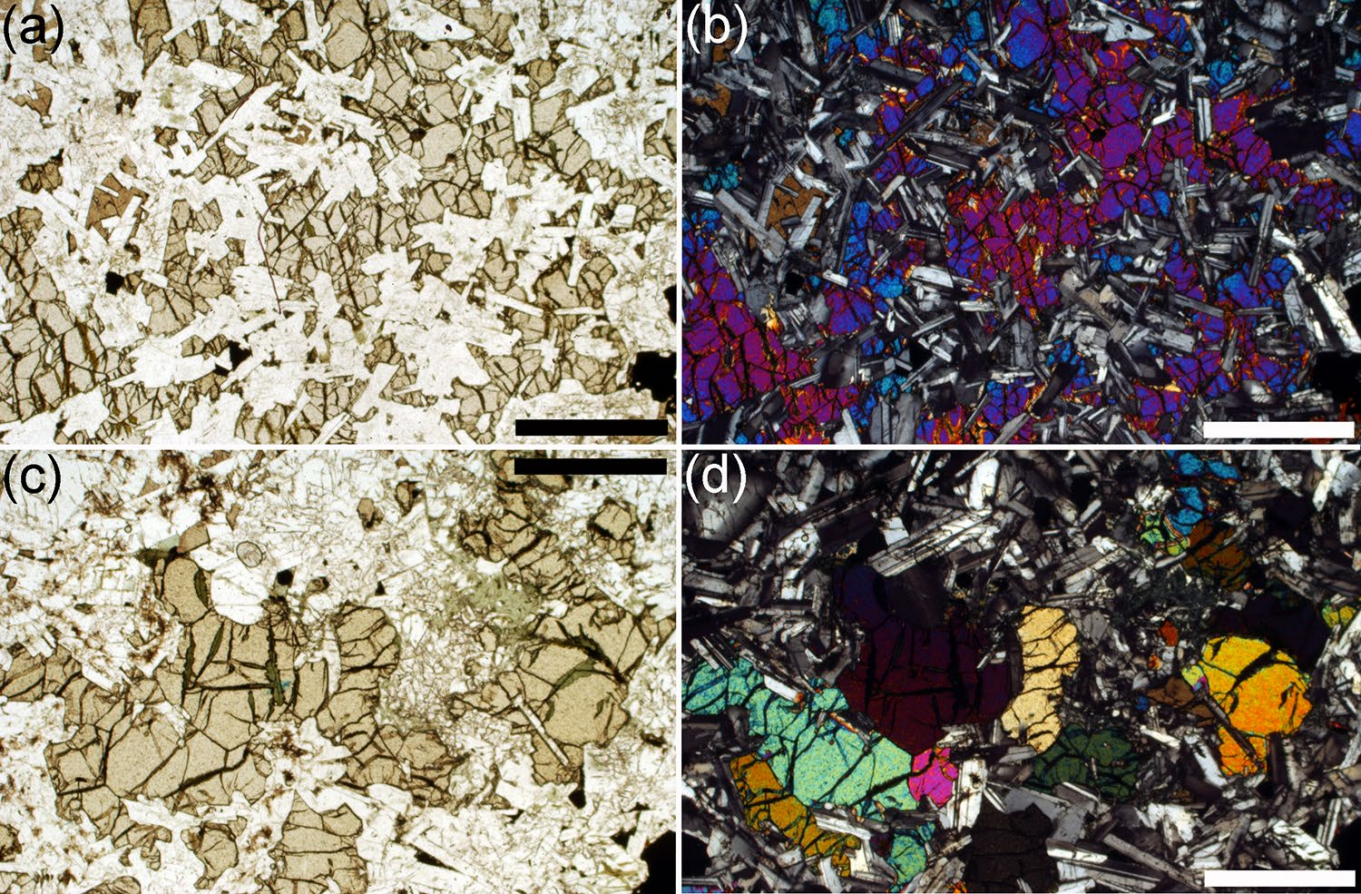
Photomicrographs of microstructures in the crinanite, with pairs of images photographed with plane polarised light and under crossed polars. **a, b** S82/68, from 120.73 m stratigraphic height. Olivine in this sample is entirely interstitial, forming extensive oikocrysts enclosing plagioclase. *Scale bar* is 1 mm long. **c, d** Sample SC1160, from 148.71 m stratigraphic height. Olivine in the upper 10 m of the crinanite is not interstitial but forms irregular clusters with an inverted microstructural progression mirroring that of the upper part of the picrodolerite. *Scale bar* is 1 mm long

The contact between the underlying Lower Picrite and the PCU is sharply defined in both outcrop and thin-section. It is marked by an abrupt increase in the range of grain sizes of the modally dominant olivine (Gibb and Henderson 1989; compare Figs. 4a, 6b, 7a). The picrodolerite at the base of the PCU contains olivine, Cr-spinel, plagioclase, interstitial (commonly poikilitic) augite, titaniferous magnetite and ilmenite. The olivine mode decreases with stratigraphic height (Gibb and Henderson 1989; Fig. 2; Table 1). This decrease is generally smooth, apart from an abrupt decrease at ~32 m which is followed by a subsequent slower rate of decline through the overlying several tens of metres of stratigraphy to the low values typical of the crinanite (Fig. 2). Gibb and Henderson (1989) place the contact between the picrodolerite and overlying crinanite between sample S81/28 at 67.30 m and sample S81/29 at 69.68 m, where the mode decreases below 15%. Olivine is generally interstitial in the crinanite, forming oikocrysts up to 5 mm across, enclosing plagioclase (Fig. 8a, b). Exceptions to this are found in sample S82/68 (120.73 m stratigraphic height) and SC1397 (129 m stratigraphic height), which contain rare clusters and a few isolated grains of olivine. The single cluster observed in a thin-section of S82/68 forms the core of an oikocryst. Clusters and isolated grains of olivine are also present in the uppermost 10 m of the crinanite (see later).

Augite is also poikilitic in the crinanite: the shape and size of the augite and olivine oikocrysts, and the grain size of the included plagioclase are similar. The crinanite additionally contains Fe-Ti oxides (dominated by magnetite) with minor analcime, apatite and zeolite (Gibb and Henderson 1996).

Cr-spinel forms isolated euhedral grains as well as inclusions in olivine in the picrodolerite (Fig. 7a). The Cr-spinel mode at the base of the PCU decreases slowly, with a significant drop at ~32 m, followed by a slower rate of decrease to background levels at ~40 m (Fig. 2; data from Gibb and Henderson 2006). Cr-spinel is extremely rare in the crinanite, being reported in only a single sample [S81/34, from 96.4 m stratigraphic height, Gibb and Henderson (1996)]. The (non-linear) co-variation of bulk rock Cr concentration and the olivine mode is shown in Fig. 5 (data from Gibb and Henderson 2006).

The volumetric ratio of plagioclase to pyroxene is high at the base of the picrodolerite, decreasing upwards to relatively uniform values in the crinanite, although there is some fluctuation most likely related to indistinct and discontinuous cm-scale modal layering in the lowermost 20 m of the crinanite (Fig. 2; data from Gibb and Henderson 1996).

The median clinopyroxene–plagioclase–plagioclase dihedral angle, Θ_cpp_, varies systematically through the PCU (Fig. 2), forming a broadly W-shaped profile with a maximum at a stratigraphic height of 65–70 m, and minima at ~31 and 153 m. Preliminary data collected from the uppermost parts of the composite sill suggest low Θ_cpp_ in the granular olivine picrodolerite (the fourth intrusive episode, marked as GOP in the stratigraphic column in Fig. 2) and high Θ_cpp_ in the olivine teschenite (the first intrusive episode, marked as white at the top of the stratigraphic column in Fig. 2). The high value in the olivine teschenite is most likely a consequence of sub-solidus textural equilibration driven by heating due to the later intrusions (e.g. Holness et al. 2012a).

**Fig. 9:**
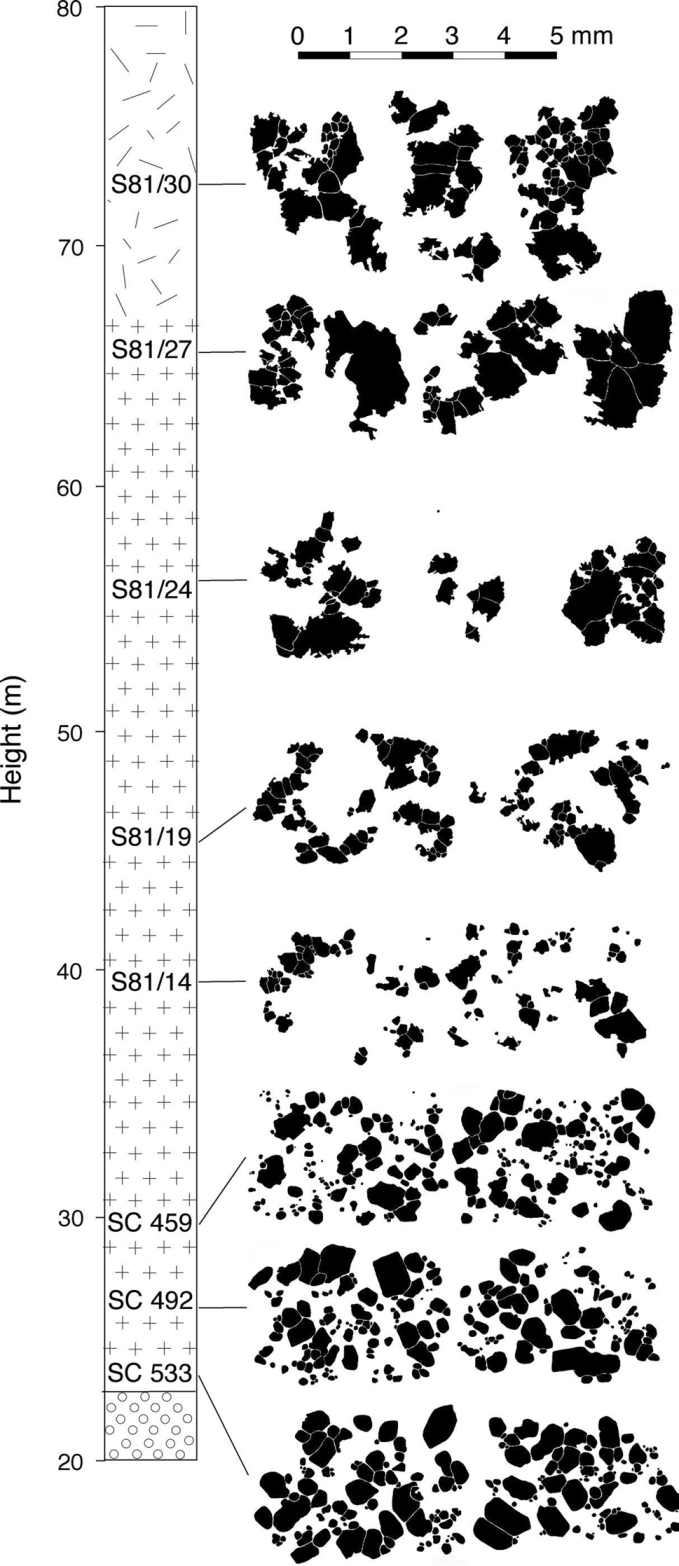
The stratigraphic change in morphology, extent of sintering, and size of olivine clusters through the picrodolerite. Each of the lowermost five samples is illustrated by the olivine grains visible in two typical photomicrographs, each 4.5 mm across. Each of the uppermost three samples is illustrated by three or more separate olivine clusters. Note the fining-upwards as a result of the loss of the coarser grains in the lowermost three samples, and the coarsening-upwards of the olivine clusters in the uppermost four samples

At the base of the picrodolerite, olivine forms abundant, rounded, unzoned, grains, with a wide range of grain sizes (Fig. 7a, b). Some isolated (at least in the plane of the thin-section), rounded grains occur, together with clusters and simple chains of rounded grains linked by small areas of grain boundary (Fig. 7a, b). Where olivine is extremely abundant, with almost all grains in contact with others, it is not possible to discern individual grain clusters, but in samples containing <20% olivine, small grains are more commonly clustered than isolated (at least within the constraints posed by 2D sectioning). Grains become clustered, with fewer isolated grains visible in thin-section, with increasing stratigraphic height. By ~40 m height, there are so few apparently isolated grains that it is likely that all such grains are, in fact, connected to a cluster outwith the plane of the thin-section. The clusters become gradually more sintered (Fig. 7c, d) with stratigraphic height, with well-developed 120° triple junctions, extensive grain boundaries and no interstitial material (Fig. 7e, f). With increasing height, the number of grains in each cluster increases, and the size of the clusters increases. The progressive morphological change in the clusters with stratigraphic height through the lower part of the PCU is shown in Fig. 9.

**Fig. 10:**
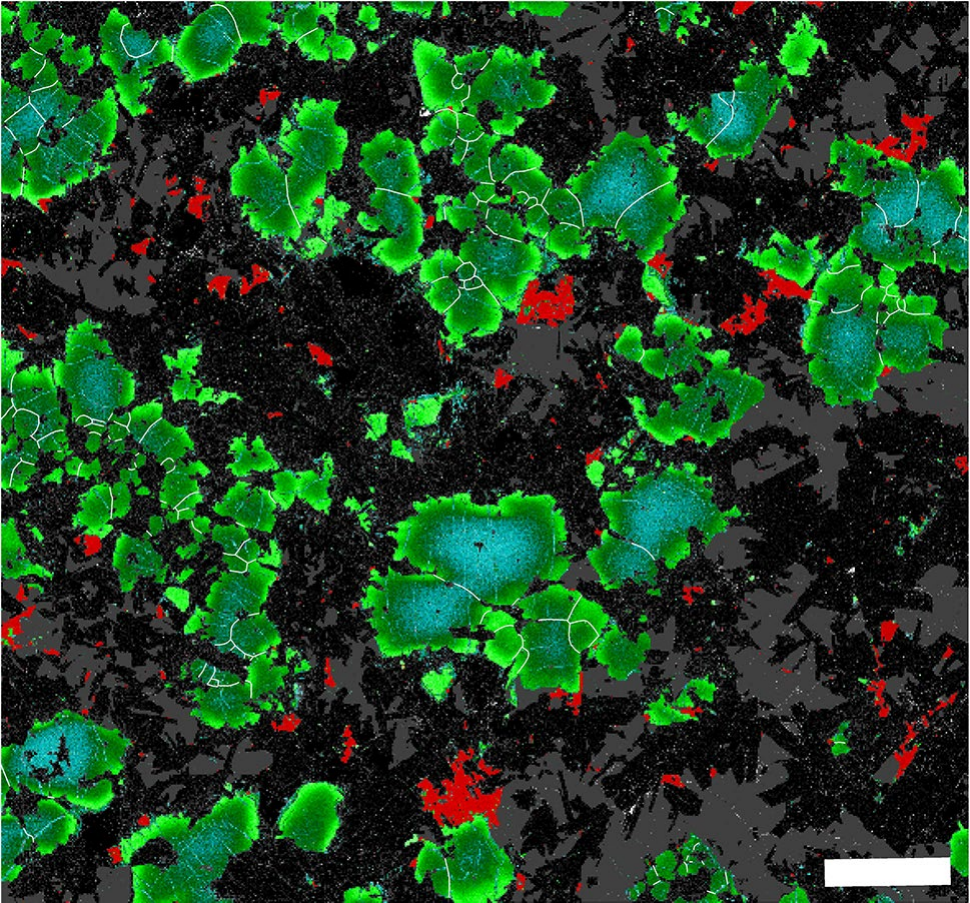
Geochemical map of sample S81/16 (stratigraphic height 41.8 m) created using QEMSCAN. The olivine grains are graded from relatively Mg-rich (*blue*) through *dark green* to *pale green* [the full range of compositions is ~Fo_73−60_, by comparison with Gibb and Henderson (1996)]. Fe-Ti oxides are shown as *red*, plagioclase is *dark speckled grey* while augite is *mid-grey*. Grain boundaries in the olivine clusters are drawn as *white lines. Scale bar* is 2 mm long

In the lower part of the picrodolerite, the average value of the ratio of the actual grain perimeter to that of a circle of the same Heywood diameter is in the range 1.07–1.16, similar to that in the picrite (Fig. 6c). However, the ratio increases with stratigraphic height, demonstrating increasing in situ overgrowth, as indicated by compositional zoning at the edges of the clusters, particularly where in contact with plagioclase (Fig. 10). Olivine clusters entirely enclosed by pyroxene oikocrysts are generally unzoned (Fig. 7g, h).

We determined average olivine grain size in the PCU, correcting the results to account for overgrowth during crystallisation of the interstitial liquid in the original accumulation. For the 5 samples in the lowest 10 m of the picrodolerite, we assumed the interstitial liquid crystallised 9 vol.% of olivine and that both olivine and plagioclase accumulated together to form a crystal pile containing 55 vol.% liquid (see later for discussion of this value). The original olivine mode, ϕ_sed_, is therefore ϕ_sed_ (vol%) = *X* − 0.55 L where *X* is the olivine mode in the fully solidified rock and *L* is the percentage of olivine that crystallised from the interstitial liquid. For the remainder of the picrodolerite, we assumed first that the amount of olivine crystallised by the interstitial liquid decreased linearly with stratigraphic height to 6 vol.% at the top of the picrodolerite, and that the accumulated pile contained 60 vol.% interstitial liquid.

**Fig. 11:**
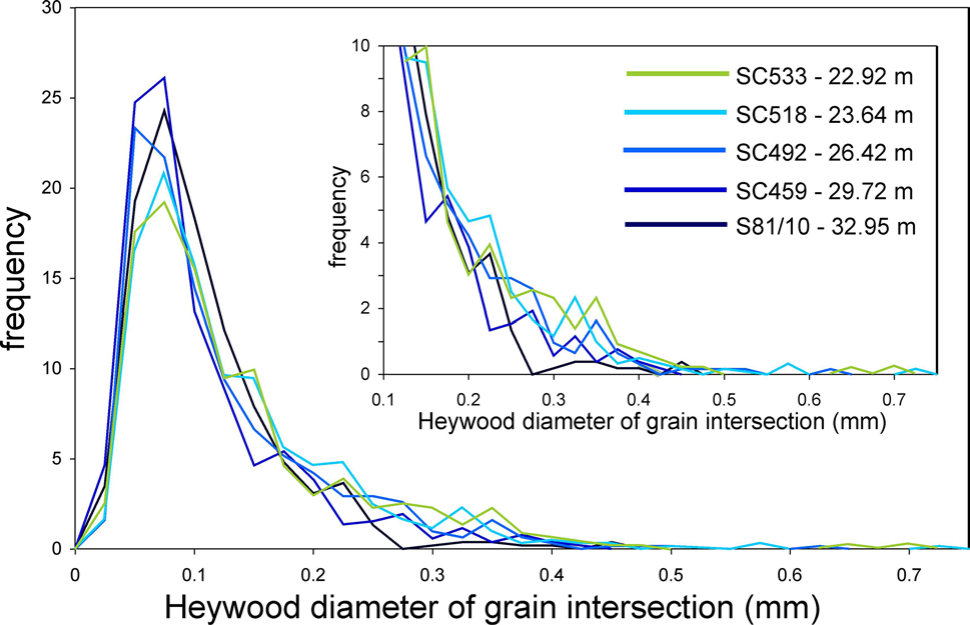
Frequency of size of individual olivine grains, as quantified by the Heywood diameter of grain intersections viewed in 2D, for the five samples comprising the fining-upwards sequence. All distributions are normalised to a population of 100. There is a gradual reduction in the proportion of large grains in the population upwards in the stratigraphy

The average olivine grain size decreases upwards (the volume-weighted mean diameters are shown in Fig. 6, though this fining-upwards is present regardless of the choice of the measure of the average diameter). A poorly defined reduction in σ suggests that this decrease is at least partly a result of the gradual loss of the larger grains (Figs. 9, 11): this type of fining-upwards progression is known as coarse-tail grading. Above ~33 m stratigraphic height, the olivine grain size increases upwards (Fig. 6a; again, this is seen for all measures of the average diameter).

A simple measure of cluster size was determined for samples above ~30 m stratigraphic height (the high olivine mode in lower samples precludes the differentiation of individual clusters) (Table 1; Fig. 6a). Note that because the clusters are highly irregular in thin-section (Figs. 7, 8, 9, 10) they may form much larger interconnected clusters in 3D than is apparent in 2D.

The average cluster size is similar to that of the average size of individual olivine grains in the lower part of the picrodolerite. Between 38.43 m and 42.40 m stratigraphic height, cluster size increases, coinciding with a morphological transition from clusters formed of loose groups of rounded grains (e.g. Figs. 3e, 7c, d, 9), to clusters that are well sintered (e.g. Figs. 7e, f, g, h, 9). Average cluster size remains constant at ~1.2 mm diameter until ~55 m height whereupon it increases to ~2 mm diameter in the highest samples that contain olivine primocrysts.

In the upper 10 m of the crinanite, non-ophitic olivine grains re-appear—this occurs between 146.2 and 148.71 m stratigraphic height. We analysed olivine grains in 5 samples from the upper 10 m of the crinanite. While they generally have irregular margins, denoting some overgrowth, the spatial variation of grain size and clustering forms a compressed and mirrored version of that in the upper part of the picrodolerite. Thus the largest and most clustered grains are seen in sample SC1160 (148.71 m stratigraphic height) (Fig. 8c, d) with grain size and extent of sintering in the clusters decreasing upwards.

In the lower part of the PCU (excepting the two samples at 120.73 and 129 m stratigraphic height), the stratigraphically highest sample in which olivine clusters are found is S81/31, at 78.85 m stratigraphic height. This is within the crinanite as defined by Gibb and Henderson (1989), but we suggest that olivine morphology, rather than olivine mode, is a better discriminant between the picrodolerite and the crinanite. We therefore suggest that in the remainder of this contribution the picrodolerite is defined as the lower part of the PCU containing compact olivine grains, while the crinanite is defined as the remainder of the PCU above 79 m stratigraphic height. The crinanite generally contains only ophitic olivine (with the exception of the upper 10 m).

**Fig. 12:**
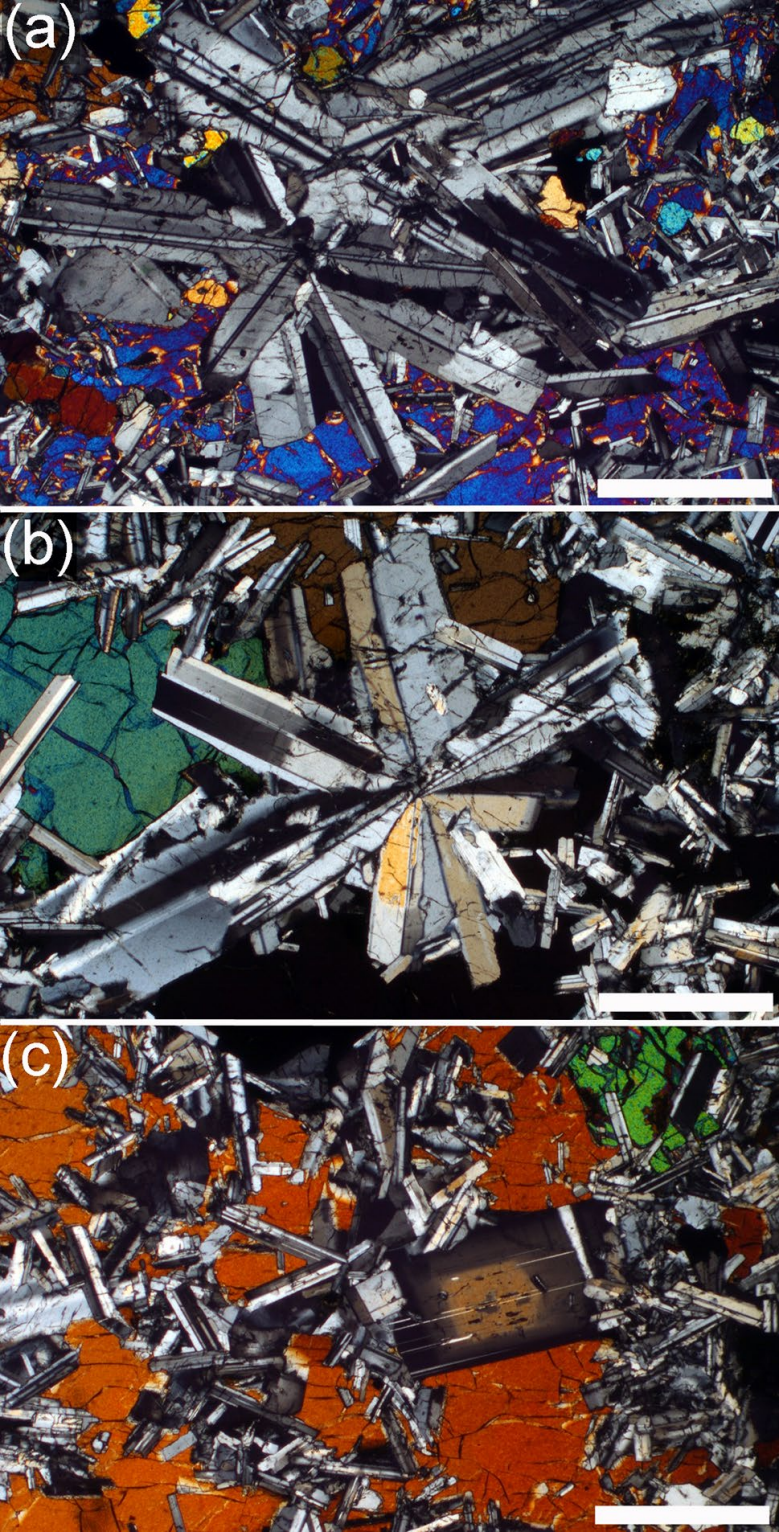
Photomicrographs of stellate plagioclase clusters and coarse-grained isolated plagioclase grains in the PCU, photographed under crossed polars. *Scale bars* in all images are 1 mm long. **a** Large stellate clusters of plagioclase in Sample S81/16 from 41.79 m stratigraphic height. Note the much larger size of the plagioclase in the cluster compared to that of the surrounding picrodolerite. **b** Sample SC1191 from 146.2 m stratigraphic height. Stellate plagioclase clusters are generally absent from much of the crinanite but re-appear with olivine clusters in the upper ~10 m. **c** Sample SC1191 from 146.2 m stratigraphic height. A large isolated plagioclase with prominent compositional zoning. Such abnormally large grains are likely to have formed part of the initial crystal cargo, along with the stellate clusters, olivine and chromite

While most of the plagioclase in the PCU forms small, randomly oriented grains, plagioclase also forms scattered stellate clusters of large elongate grains (Fig. 12a, b), some of which contain abundant melt inclusions (Fig. 12a): these formed part of the crystal load of the incoming magma (Gibb and Henderson 1996, 2006). Stellate clusters like these are common in magmas and probably form under conditions of inhibited nucleation caused by a period of superheat (e.g. Arzilli and Carroll 2013).

The clusters are not abundant, with a maximum of 2 intersected by any one thin-section. They form two types, the first comprises only plagioclase while the second contains coarse euhedral/subhedral olivines (F.G.F. Gibb, pers. commun., 2014). Commonly associated with the stellate clusters are apparently isolated grains of unusually large plagioclase, commonly containing fluid inclusions and/or distinct breaks in compositional zoning (Fig. 12c). In our sample suite, the stratigraphically lowest stellate plagioclase cluster occurs in S81/12 at stratigraphic height 35.1 m. Stellate clusters are common through the next 10 m of stratigraphy and are present, though not common, up to 80 m stratigraphic height. Apart from three samples in the range 122–129 m stratigraphic height, which contain a few unusually large, isolated, grains of plagioclase with sharply defined marginal zoning (i.e. not organised into stellate clusters), the only other sample of our suite to contain clearly identifiable stellate clusters is SC1191 at 146.2 m stratigraphic height (i.e. below the uppermost part of the crinanite where olivine is no longer ophitic). Sample SC1191 also contains a few unusually large, zoned, plagioclase grains (Fig. 12c).

The stratigraphic distribution of the stellate plagioclase is thus similar to that of the olivine phenocrysts, with a lower zone rich in clusters overlain by a cluster-free zone followed by an uppermost cluster-bearing zone. However, the stellate cluster distribution is offset to higher levels at the base, and to lower levels at the top, of the PCU.

### Summary

The PCU can be subdivided into four distinct regions (Fig. 2). The lowermost two subdivisions correspond approximately to the picrodolerite of Gibb and Henderson (1996) and comprise a lower ~10 m-thick subdivision characterised by fining upwards of the olivine phenocryst load and a relatively high bulk rock Cr content (Fig. 2), and an upper ~45 m-thick subdivision characterised by coarsening upwards of the olivine phenocrysts and clusters, an upwards increase in the extent of sintering of clusters of olivine grains, and an increase in the extent to which individual grains depart from a rounded shape. The base of this upper subdivision is gradational, involving a gradual change from fining-upwards to coarsening-upwards, with a step-wise increase in cluster size: we place it at 33 m. The top of the upper subdivision, at 79 m stratigraphic height, is marked by the loss of compact olivine grains. The third and fourth subdivisions form the crinanite, with a lower part characterised by an absence of olivine phenocrysts (with rare exceptions), and an upper 10 m in which olivine phenocrysts re-appear. Olivine morphology in the upper 10 m of the crinanite mirrors that in the upper part of the picrodolerite, suggesting that these two subdivisions formed contemporaneously, one growing up from the floor and the other down from the roof. The position of the most evolved rocks, at 125–130 m (Gibb and Henderson 1996, 2006) marks the Sandwich Horizon.

## Discussion

### Is the Shiant Isles Main Sill a composite intrusion?

An issue which must be resolved before a full discussion of the new observations is whether the Shiant Isles Main Sill is a composite intrusion, as advocated by the work of Gibb and Henderson, or whether the stratigraphic heterogeneities are a result of the inflation of the sill by a single batch of magma of progressively changing composition followed by in situ fractionation processes, as suggested by Latypov (2014).

As Gibb and Gibson (1989) point out, the continuity of columnar jointing through the entire body points to its assembly over a sufficiently short time to permit the intrusion to cool as a single entity. A more detailed picture of the cooling history can be obtained from the stratigraphic variation of the dihedral angle, Θ_cpp_ (Fig. 2; Table 2), which maps directly onto the time taken to crystallise (Holness et al. 2012b). The maximum value of Θ_cpp_ occurs in the lower part of the PCU, close to the mid-point of the entire intrusion, denoting that this horizon was that which cooled the slowest, consistent with the observation of continuous columnar jointing through the entire body. However, the decrease in Θ_cpp_ towards both margins of the PCU, particularly the steep decrease towards the base of the PCU, shows that this part of the sill was a distinct intrusion, in agreement with the work of Gibb and Henderson: had the entire body been a single, 168 m thick, intrusion the decrease in Θ_cpp_ in the PCU would have been more symmetrical. The reversal in Θ_cpp_ close to the base of the picrodolerite, and a less well-defined reversal at the top of the crinanite, are features commonly present at the margins of thick sills. They are a consequence of sub-solidus textural adjustment of a relatively fine-grained marginal zone (Holness et al. 2012a, b). The well-developed nature of this reversal at the base of the PCU is consistent with annealing caused by heat released from the Lower Picrite.

**Table 2:** Dihedral angle data for the Shiant Isles Main Sill

Sample	Stratigraphic height (m)	*n*	Θ_cpp_	SD
SC518	23.63	80	83.5 ± 3	14.5
SC492	26.42	80	82 ± 2	15.3
SC448	27.48	100	82 ± 2.5	14.0
S81/8	30.51	80	81 ± 2.5	14.5
SC409	32.46	100	81 ± 2.5	13.8
S81/12	35.08	100	83 ± 1.5	11.7
SC380	36.68	100	83 ± 2.5	13.0
SC339A	40.29	100	88 ± 2	13.1
S81/16	41.79	100	88 ± 2.5	12.3
S81/22	52.88	100	89.5 ± 2	11.1
S81/27	65.87	100	91 ± 3	14.1
SC8	72.39	100	91 ± 1	12.4
S81/32	80.80	100	90.5 ± 1.5	10.1
S81/34	96.65	100	90 ± 1.5	13.4
S81/35	100.86	100	90 ± 2	15.6
S82/61	110.3	100	88 ± 3	13.8
SC1464	125.02	100	87 ± 3	12.4
S82/69	132.92	100	85 ± 3	17.3
SC1160	148.71	100	84 ± 3	15.3
SC1132	152.58	100	83 ± 2.5	15.0
SC1091	155.26	80	86 ± 2.5	11.8
SC1081	156.16	100	87 ± 2	13.4
SC1072	157.29	80	88 ± 4	12.7
SC1054	159.43	100	80.5 ± 1	15.0
S82/80	164.88	80	90.5 ± 3	11.3

The stratigraphic heights are those of Gibb and Henderson (1996). The number of angles measured is given by *n*. The median clinopyroxene–plagioclase–plagioclase dihedral angle is given by Θ_cpp_, with the uncertainties calculated according to Stickels and Hücke (1964). The standard deviation is given by SD

Further evidence for the composite nature of the intrusion is provided by the very different populations of olivine grains in the Lower Picrite and the base of the picrodolerite, with a distinct change in the degree of sorting at the boundary (Fig. 6), and the absence of stellate plagioclase from the Lower Picrite. We conclude that the Lower Picrite and the PCU represent the intrusion of two distinct magmas and that therefore, in agreement with the work of Gibb and Henderson, the Shiant Isles Main Sill is a composite intrusion.

### The Lower Picrite: an unmodified crystal-rich slurry?

The critical observations that we can use to understand the history of the Lower Picrite are the D-shaped profile of olivine mode (Gibb and Henderson 1992, 1996), the narrow range of grain size (Fig. 6), the increase of average grain size towards the contact with the overlying PCU (Fig. 6), and the linear correlation between bulk rock Cr concentration and the olivine mode (Fig. 5).

The distribution of grain sizes in igneous rocks is commonly used to make inferences about the nucleation and growth kinetics during solidification (e.g. Marsh 1998). However, the D-shaped profile of the olivine mode suggests that the distribution of olivine grain sizes in the Lower Picrite is likely to have been modified from the original by sorting during magma transport, particularly by grain dispersive pressures that concentrated the olivine crystal load in the centre of the inflating picrite sill (Gibb and Henderson 1992).

The preservation of the D-shaped profile of the olivine mode, together with the close correspondence of olivine mode with the bulk rock Cr concentration, shows that there was no density sorting of the crystal cargo, consistent with minimal, post-emplacement, gravitational settling (Gibb and Henderson 1992). The forces acting on suspended particles in a laminar flow are a function of grain size, resulting in the largest grains migrating to the intrusion centre (Komar 1972a, b)—the observed upwards increase of average grain size in the Lower Picrite towards the contact with the overlying PCU suggests that the largest grains in the picrite intrusion were found towards the top of the original 24 m-thick body.

The solidification time of a sill of thickness *h* is given by ${h}^{2}/4\lambda \kappa$, where λ = 0.788 is given by the solution to ${S}^{-1}=\lambda \sqrt{\pi }{e}^{{\lambda }^{2}}\left(1+\mathrm{erf}\left(\lambda \right)\right)$which may be derived from the Stefan condition at the edge of the sill (Turcotte and Schubert 2002). Here $S=L/{c}_{p}=0.22$ is the Stefan number, $L=320$ kJ kg^−1^ is the latent heat, c_p_ = 1.2 kJ kg^−1^ K^−1^ is the specific heat, Δ*T* = 1200 K is the temperature difference between the sill and the country rock and κ = 10^−6^ m s^−2^ is the thermal diffusivity, giving a characteristic timescale for cooling of the 25 m-thick picrite of ~2 years.

Olivine in the Lower Picrite is ~Fo_80_ (Gibb and Henderson 1996), of density ~3300 kg m^− 3^. Using the suggested composition for the parental magma of Gibb and Henderson (2006), and assuming an intrusion temperature of 1200 °C (Gibb and Henderson 2006), a confining pressure of 0.5 kbar and a H_2_O content of 1 wt%, the parental magma had a density of 2600 kg m^−3^, calculated according to the method of Bottinga and Weill (1970), using volume and thermal expansion data from Lange and Carmichael (1987) and Kress and Carmichael (1991). This leads to a density difference, ∆ρ, between the olivine and the carrier liquid of 700 kg m^− 3^. From Giordano et al. (2008) the composition for the parental magma (Gibb and Henderson 2006) leads to a predicted viscosity of 2 Pa s. The Stokes settling velocity of an olivine grain of diameter 0.5 mm is ~5 × 10^− 5^ m s^− 1^, enabling the grain to settle 25 m in a week, a much shorter timescale than that of cooling. Settling was clearly ineffective in the Lower Picrite. This may have been achieved by a prolonged or episodic series of injections of crystal-rich magma (e.g. Hayes et al. 2015), or because the volume of suspended crystals was sufficient to create a relatively rigid framework throughout the sill very soon after injection.

### The role of crystal settling, growth and clustering in the evolution of the PCU

The difference in olivine morphology between the lower 10 m and the remainder of the picrodolerite is of fundamental importance in decoding the history of the intrusion. It is possible that the stratigraphic variation in microstructure resulted from the crystallisation of successive injections of magma containing populations of olivine phenocrysts with a progressive change in the size of clusters and the extent of sintering (c.f. Marsh 2013, 2015). However, the smooth morphological variation through the PCU, and the absence of sharp internal contacts, make it more likely that the change from a fining-upwards accumulation of individual or weakly clustered olivine grains to a coarsening-upwards sequence of highly clustered olivine grains took place within the sill itself: we therefore base the remainder of the discussion on this assumption.

The total thickness of the PCU which contains olivine clusters (i.e. the coarsening-upwards sequence plus the 10 m at the top of the crinanite) is ~55 m, of which 82% is found at the base. The position of the most evolved bulk compositions in the PCU at 125–130 m stratigraphic height (Gibb and Henderson 2006) suggests that the crystal mushy layer (or solidification front) propagating upwards from the floor comprised 75–80% of the total thickness of the PCU. The similarity of these proportions suggests that the ratio of crystal accumulation rates on the floor and roof of the PCU was similar during most of its solidification history. Moreover, the strong asymmetry of accumulation rates at the floor and roof cannot have been the consequence of in situ crystallisation in solidification fronts, with no subsequent movement of crystals, because the PCU cooled significantly more slowly through its floor compared to the roof (Fig. 2). Therefore, much of the material accumulating on the floor must have been derived from elsewhere.

#### Identifying the original crystal load in the incoming magma

In the absence of significant crystal growth or particle coarsening by agglomeration/synneusis, settling of a polydisperse crystal load will always result in a fining-upwards sequence. This is well understood to be the case for settling from a static magma (Mirza and Richardson 1979; Selim et al. 1983; Davis and Acrivos 1985), and the same also holds for settling from a convecting magma. Martin and Nokes (1988) showed that crystals can settle from a vigorously convecting magma, even when the convective velocity is much greater than the Stokes settling velocity of the crystals, because they can escape (at their Stokes velocity) when they enter the basal stagnant boundary layer (see the more detailed discussion in the following section). The Martin and Nokes (1988) model is typically applied to a monodisperse crystal population and predicts that the concentration of crystals remaining in the liquid falls exponentially with time. If we extend their reasoning to a system containing a polydisperse crystal population, since bigger particles settle more quickly in the stagnant layer, the bigger particles are removed from the bulk magma more quickly than small grains, leading to the accumulation of a fining-upwards sequence on the floor.

For this reason, the olivine in the picrodolerite must have accumulated during two distinct stages: if the large grains and clusters now present in the upper part of the picrodolerite were brought into the inflating sill as part of the original crystal load, gravitational sorting during settling would have concentrated them at or near the base of the picrodolerite, regardless of whether the magma was static or convecting. The corollary of this is that the olivine in the coarsening-upwards sequence in the upper part of the picrodolerite was not present in the incoming magma. The original crystal load therefore is represented by the fining-upwards sequence in the basal 10 m. The corrected olivine modes (i.e. with overgrowth removed) in the basal 10 m of the picrodolerite therefore suggest that the incoming magma carried a load of 3 vol.% olivine, not the 10 vol.% suggested by Gibb and Henderson (2006).

Gravitational sorting of particles can occur while they are being transported in a current moving by laminar flow (Choux et al. 2004). The Reynolds number of magma of density 2.6 g cm^− 3^, viscosity 10 Pas, flowing in a 130 m planar-sided conduit, will only be sufficiently low for laminar flow (i.e. below ~10^3^) for flow velocities <0.03 m s^− 1^. Such low flow rates are unlikely for such a large, laterally extensive intrusion [see, for example, Kavanagh et al. (2006) and Rubin (1998)] and are much lower than comparable recent seismically measured events for a much smaller intrusion (Sigmundsson et al. 2014). Flow of the incoming magma was therefore likely to have been turbulent, so the gravitational sorting we observe in the lower 10 m of the PCU occurred only once the sill was fully inflated.

The most straightforward understanding of settling is based on the assumptions that the magma was static and that settling was not perturbed by the presence of other particles. The inference of a 3 vol.% olivine load ensures that interactions between particles can be neglected (Happel and Brenner 1965). An additional requirement is that the initial particle concentration was sufficiently low to prevent the modification of settling behaviour by the upwards counterflow of displaced liquid (Druitt 1995; Schwindinger 1999).

For such a case, Farr et al. (2016) showed that the rate of build-up of a settled layer, $\dot{H}$, is approximated by:

$$\dot{H}=\frac{{{\phi }_{\text{susp}}}}{{{\phi }_{\text{sed}}}}u=\frac{{{\phi }_{\text{susp}}}g\Delta \rho }{18{{\phi }_{\text{sed}}}\eta }D_{3,1}^{2}$$

where *u* is the Stokes settling velocity, ∆*ρ* is the density difference between olivine and the magma, *η* is the magma viscosity, and ϕ_susp_ is the volume fraction of olivine in the incoming magma. Although clustering and the formation of irregular chains will result in a departure from Stokes’ Law behaviour (since high aspect ratio particles fall more slowly than spherical particles), for aspect ratios up to 4 the reduction in settling velocity is of order 10% (Kerr and Lister 1991).

As argued earlier, the difference in density, ∆ρ, between the olivine in the basal picrodolerite and the parental magma is 700 kg m^−3^. From Giordano et al. (2008), the two compositions for the PCU parental magma (Gibb and Henderson 2006) lead to predicted viscosities in the range 3.9–8.5 Pa s. Using the values for D_3,1_ given in Table 1 for the five samples for which we have data, we can use a step-wise integration to constrain the time taken to form the 10 m-thick fining-upwards settled layer of olivine in a static magma to be 22–48 weeks.

For comparison, we calculated the distance moved by a solidification front propagating upwards from the floor of a 135 m-thick sill intruded at 1200 °C into country rock at 0 °C, using the relationship of Holness et al. (2012b). Accounting for the latent heat of solidification, and assuming both diffusive heat loss and that solidification is complete by 1000 °C, only 2.6–3.9 m of solidification will have occurred on a timescale of 22–48 weeks: these are overestimates of the upwards progress of the solidification front because cooling at the floor of the PCU was slowed by the underlying Lower Picrite. We can conclude from this that initial settling of the crystal load would have occurred rapidly relative to the upwards movement of the solidification front, forming an essentially isothermal pile on the sill floor, with negligible associated in situ crystallisation.

#### Did the olivine clusters grow in situ at solidification fronts?

If we now consider the olivine in the remainder of the picrodolerite, a further argument against it being part of the incoming crystal load can be constructed by considering the particular case of the olivine at the roof of the PCU. Although Gibb and Henderson (1996) suggested that it formed part of the original crystal cargo, consideration of cluster settling velocity relative to the propagation of the solidification front precludes this. Clusters 2 mm in diameter are present in sample SC1160 at 148.7 m stratigraphic height, some 10 m from the roof of the PCU. Their settling velocity is 2–4 × 10^− 4^ m s^− 1^, enabling them to sink 25 m in a day, considerably faster than any plausible propagation rate for the roof solidification front. A possible way round this problem is if we hypothesise that the olivine clusters currently at the roof nucleated and grew *in situ* at a downwards-propagating solidification front. The corollary that the clusters on the floor must also have grown at a solidification front, either at the roof (with subsequent detachment and accumulation on the floor) or at the floor itself.

Clusters grown in situ would necessarily have formed by what is variously termed secondary nucleation (Melia and Moffitt 1964; Brown 1972), auto-nucleation (Brown 1972), self-nucleation (Campbell 1978), or sympathetic nucleation (Aaronson et al. 1995). Such a mechanism involves the heterogeneous nucleation of mineral grains on an existing grain of the same phase, and is well documented for plagioclase (e.g. Kirkpatrick 1977) but not for olivine. One might then reasonably suggest that the increase in the size of both clusters and their constituent grains observed in the picrodolerite, together with the increase in the numbers of grains in each cluster and the extent of sintering of each cluster (e.g. Schwindinger 1999), is a consequence of a gradually reducing cooling rate and an increase in the time available for nucleation and growth.

A significant problem with the hypothesis of in situ cluster growth is that it requires that the volumetric proportion of olivine crystallising from the bulk liquid decreased with time through the formation of the upper part of the picrodolerite (with different proportions growing at the roof and floor), and then remained constant during the crystallisation of the crinanite: this is implausible. The hypothesis is also inconsistent with the asymmetry of cluster distribution at the base and top of the PCU (since clusters grown at the roof would have required the same time to form as those on the floor even though they are much closer to the boundary of the sill). The compositional zoning in the clustered olivines (Fig. 10) is also inconsistent with the progressive nucleation of additional grains on the margins of clusters; if this was the case, we would expect grain centres to be progressively more evolved towards the margins of the clusters, but the pattern (visible in 2D) is more irregular than this. While the larger grains generally have the most primitive cores, small grains apparently in the centres of clusters do not have primitive cores. We conclude therefore that the olivine in the coarsening-upwards part of the picrodolerite and in the upper 10 m of the crinanite cannot have grown at solidification fronts.

#### Comparison of settling from static and convecting magma

A solution to this conundrum can be obtained, if we consider the possibility that the magma in the PCU convected during olivine growth and accumulation. To assess the likely consequences, we develop the original Martin and Nokes (1988) theory for the sedimentation of a dilute suspension of monodisperse crystals from a strongly convecting magma to examine the effect on a polydisperse grain distribution.

If we assume the particle concentration is always sufficiently low to prevent particle interactions, it is well understood that Stokes’ settling in a static (and initially well-mixed) magma leads to the settling of individual crystals at a constant rate. Each size class is deposited at a constant rate, until all the grains of that size class have fallen out of suspension, leading to the abrupt and complete disappearance of progressively smaller size classes upwards in the accumulation. This is not the case for settling from a convecting magma.

Following Martin and Nokes, we ignore the possibility of re-entrainment of previously settled material and assume no grain growth of the suspended particles. The theory posits that there are *N* crystals per unit volume in a magma chamber of height *h*, with a roughly uniform concentration everywhere, including at the base of the convecting zone where it transitions to the stagnant boundary layer. In this stagnant layer, the crystals sediment at the Stokes velocity *u*, so that the concentration falls according to the relation $h\dot{N}=-uN$ and the half-life of the suspended crystals is therefore *h*/*u*.

Here, we consider a distribution of different sized crystals in suspension, where $N\left(D,t\right)dD$ is the number of crystals per unit volume at time $t$ with characteristic sizes between $D$ and $D+\mathrm{d}D$. The mass of crystals in suspension per unit area of chamber floor is thus

$$M=\underset{0}{\overset{\infty }{\mathop \int }}\,\rho \frac{\pi }{6}{{D}^{3}}hN\left( D,t \right)\text{d}D,$$

where $M\left(t\right)$ is the total mass of crystals. For low total initial crystal number densities, settling of individual crystals is given by the Stokes settling velocity which depends on the crystal diameter,

$$u\left( D \right)=~\frac{\Delta \rho ~g~{{D}^{2}}}{18~\eta }$$

where we assume both that the density difference between crystal and fluid, ∆*ρ*, and the fluid viscosity, *η*, are constant. We follow Martin and Nokes (1988) and also assume that the convective fluid velocities are typically much greater than the Stokes settling velocity and hence the distribution of crystals remains uniform within the fluid. Settling only occurs within the viscous boundary layer at the base of the chamber, where fluid velocities become negligible. The mass of crystals that sediment through the stagnant viscous boundary layer per unit time is therefore given by

$$\frac{\text{d}M}{\text{d}t}=-\underset{0}{\overset{\infty }{\mathop \int }}\,\rho \frac{\pi }{6}{{D}^{3}}u\left( D \right)N\left( D,t \right)\text{d}D.$$

From this, and given an initial crystal distribution, the time scale for sedimentation, and the variation of the mean crystal diameter, can be calculated. To make analytical progress, and to illustrate the inevitable consequences of such a model, we assume an initial Maxwell-type distribution of suspended crystals,

$${{N}_{\text{susp}}}(D,t)=A{{D}^{n}}\exp [-{{(D/{{D}_{0}})}^{2}}],$$

where ${D}_{0}$ and $n$ are parameters of the distribution which set the mean and variance. The argument of Martin and Nokes (1988), applied to each size of crystal, suggests that the crystal distribution remaining in suspension varies with time and is given by

$${{N}_{\text{susp}}}\left( D,t \right)={{N}_{\text{susp}}}\left( D,0 \right)\exp \left[ -\alpha {{(D/{{D}_{0}})}^{2}}t \right],$$

where $\alpha =\Delta \rho g \; {D}_{0}^{2}/18\eta h$ is the inverse time for a crystal of diameter ${D}_{0}$ to settle a distance $h$. Thus the crystal distribution as a function of time is given by

$${{N}_{\text{susp}}}\left( D,t \right)=~A{{D}^{n}}\exp \left[ -{{\left( \frac{D}{{{{\tilde{D}}}_{0}}\left( t \right)} \right)}^{2}} \right].$$

An immediate result of this analysis is that the average size of the suspended crystals decreases with time

$${{\tilde{D}}_{0}}\left( t \right)=\frac{{{D}_{0}}}{\sqrt{1+\alpha t}}.$$

Thus the crystal content of the cumulate pile should fine upwards, as we argued in outline form earlier. The volume of crystals in suspension over unit area of floor at any time is given by

$${{V}_{\text{susp}}}\left( t \right)=\underset{0}{\overset{\infty }{\mathop \int }}\,h\frac{\pi }{6}{{D}^{3}}{{N}_{\text{susp}}}\left( D,t \right)\text{d}D=\frac{\pi Ah}{12}\Gamma \left( 2+\frac{n}{2} \right)\tilde{D}_{0}^{n+4},$$

and so the volume deposited in the cumulate pile can be estimated, ${V}_{\mathrm{c}\mathrm{u}\mathrm{m}\mathrm{u}\mathrm{l}\mathrm{a}\mathrm{t}\mathrm{e}}\left(t\right)= {V}_{\mathrm{s}\mathrm{u}\mathrm{s}\mathrm{p}}\left(0\right)-{V}_{\mathrm{s}\mathrm{u}\mathrm{s}\mathrm{p}}\left(t\right)$. If we assume that the thickness of the cumulate pile is proportional to the total volume of crystals deposited as a function of time, then the thickness of the pile increases as

$${{h}_{\text{cumulate}}}\left( t \right)={{h}_{\max }}\left[ 1-{{(1+\alpha t)}^{-(n+4)/2}} \right],$$

where again the timescale for growth of the cumulate pile is

$$\tau =~\frac{1}{\alpha }=\frac{18\eta h}{g~\Delta \rho ~D_{0}^{2}}$$

This result shows that the flux of particles to the accumulated pile on the floor decreases with time. Moreover, grains in a convecting magma settle in proportion to their concentration and settling velocity. This means that, for the case analysed here (and in contrast to the case for settling from static magma), the shape of the size distribution remaining in the bulk magma does not change for the sample distribution shown here, but is progressively shifted to smaller grain sizes.

#### Did the PCU magma convect?

Comparison of frequency distributions for grain intersection size (reported as Heywood diameter) demonstrates that the proportion of large grains present in each of the five samples forming the fining-upwards sequence progressively decreases through the sequence (Fig. 11). This preliminary observation is consistent with settling from a convecting magma, and will be fully explored in a future contribution.

Experimental work demonstrates that a significant particle load can act to damp convection in a system cooled from above, with olivine concentrations as low as 0.5 wt% damping convection in a basaltic system (Koyaguchi et al. 1993). Although the forces driving convection in the PCU are more complex than those in the experiments of Koyaguchi et al. (1993), it is possible that the crystal load was initially too high to permit vigorous convection. A close look at the stratigraphic variation of bulk rock Cr concentration demonstrates that almost all the Cr-spinel was deposited on the sill floor by ~30 m height. This highly efficient settling of such small grains (with a corresponding low settling velocity, e.g. Campbell et al. 1978) is most likely because Cr-spinel grains tend to adhere to olivine phenocrysts (Fig. 3), forming composite particles of greater density and therefore with a greater settling velocity than either individual olivine or spinel grains. If we assume there is no relationship between grain size and the mass of Cr-spinel adhering to each olivine grain, the abrupt decrease in Cr content at ~30 m may suggest that settling took place under essentially static conditions. In contrast, the subsequent slow decline of Cr content to background values is suggestive of the gradual decrease in accumulation expected for settling from a convecting magma. This progression is consistent with the initial damping of convection by a high crystal load, with convection beginning once sufficient floor accumulation had begun, reducing the suspended load. It is also consistent with the gradational change from the fining-upwards sequence to the coarsening-upwards sequence. However, this interpretation is dependent on only a few data points from the base of the PCU, which may have been affected by sorting during the initial inflation stage of the sill’s history.

Following our earlier arguments, the presence of large olivine clusters some 10 m from the top of the crinanite can only be explained by convection. We suggest that olivine clusters formed by the progressive growth and aggregation of the finest grains that remained in the bulk magma after the bulk of the crystal cargo had settled to the floor. These small remaining crystals grew as the magma cooled, but remained suspended by convection, gradually forming clusters as convective motion of the magma brought them into contact with other grains (e.g. Schwindinger 1999) by a process variously referred to as synneusis (Vance 1969; Schwindinger and Anderson 1989), agglutination (Nespolo and Ferraris 2004) or aggregation, followed by sintering. This process requires crystals to move relative to each other (Vance 1969; Smith 1974; Nespolo and Ferraris 2004), and is facilitated for crystals suspended in a convecting magma compared to those in a stagnant magma. The process of aggregation of initially isolated grains by synneusis followed by sintering results in characteristic compositional zoning patterns (Schwindinger and Anderson 1989; Jerram et al. 2003), similar sizes of grains (Schwindinger and Anderson 1989), differences in composition between grains within a cluster (Schwindinger and Anderson 1989), and a preferred relative orientation between adjacent grains (due to the greater likelihood of bonding at low energy grain boundaries (Vance 1969) or the development of preferred orientations in a flow (Schwindinger 1999)). These features are present in the Shiant clusters.

Those grains on the edges of the clusters could continue to grow from the bulk magma and this, together with continued capture of olivine crystals, resulted in a progressive increase in cluster size. Such a pattern of stochastic meeting and clustering is consistent with the random nature of the core compositions in the clusters (Fig. 10), although a temporal sequence is visible in that large grains tend to have the most primitive cores while the small ones are more evolved. Clusters that escaped the convecting magma and settled to the floor created a coarsening-upwards succession. While our treatment of settling from a convecting magma takes no account of a temporal size increase of the suspended particles, this idea is much like the suggestion of Bowen (1915), who attributed reverse size sorting in his settling experiments to the uppermost crystals having had longer to grow during the longer time they took to settle from higher in the charge. Some clusters brought to the roof of the sill by upwards-moving convection currents were entangled and trapped in the irregular margin of the upper solidification front. The relatively low mode of olivine in this upper 10 m is a consequence of the low probability of such trapping. We conclude that, in contrast to the fining-upwards sequence, which might have formed by settling from a convecting magma, the olivine in the remainder of the picrodolerite can only have settled from a convecting magma.

### The development of the PCU by settling from a convecting magma

#### Formation of the fining-upwards sequence

The olivine mode at the base of the picrodolerite is 48.5 vol.% which, when we take into account overgrowth from the interstitial liquid, corresponds to an original olivine volume fraction of ~43 vol.% in the settled accumulation. This is lower than the 56–54 vol.% expected for a random loose packing of cohesionless monodisperse spheres (defined as the loosest, mechanically stable packing state; Onoda and Liniger 1990; Ciamarra and Coniglio 2008; Zamponi 2008; Farrell et al. 2010), and lower still than random loose packings achieved for polydisperse particles (Epstein and Young 1962; Jerram et al. 2003). However, the efficiency of random loose packing depends on particle cohesion: for strongly cohesive particles, a stable distribution can be achieved at volume fractions <55% (Dong et al. 2006; Yang et al. 2007), and the presence of highly non-spherical, loose clustered chains of olivine will reduce this still further (Campbell et al. 1978; Jerram et al. 2003). Random loose packing is also dependent on the viscosity of the liquid, with more open packings expected for particles in a viscous liquid (Delaney et al. 2011). If we assume that compaction was insignificant, and that the crystal pile was not densified by shear or shaking, it is therefore plausible that the basal part of the settled crystal load comprised an olivine-dominated, randomly loose packed, mechanically stable framework of crystals with a solid fraction of ~44% (including 1 vol.% Cr-spinel).

The (pre-overgrowth) volume fraction of the settled olivine and spinel decreases to ~25 vol.% at the top of the fining-upwards sequence. While a combination of strong grain cohesion, high liquid viscosity, and the presence of elongate and irregular loose clusters might make it possible to create a random loose packing at such low volume fractions (Dong et al. 2006; Yang et al. 2007; Delaney et al. 2011), it is unlikely that the observed reduction in olivine mode reflects a reduction in the solid fraction required to create a mechanically coherent accumulation. Instead, we suggest that the solid fraction of the crystal pile was likely to have been constant (at ~44 vol. %) over 10 m, with the reduction in olivine matched by a corresponding increase in the mode of another phase accumulating simultaneously.

The incoming magma contained a small amount of plagioclase phenocrysts of composition ~An_84_ (Gibb and Henderson 1996) and density ~2730 kg m^−3^, greater than that (2600 kg m^−3^) of the carrier liquid. The relatively high plagioclase:augite ratio in the lower 10 m of the picrodolerite (Fig. 2) is consistent with at least some of these incoming plagioclase phenocrysts having settled to the floor.

We can thus interpret the modal variations in the lower 10 m of the picrodolerite as a consequence of gravitational sorting of the original crystal load, with a lower portion containing those olivines whose settling velocity was increased by included or adhering Cr-spinel grains, grading upwards to a Cr-spinel-poor olivine-rich zone and further upwards into an increasingly plagioclase-rich (and Cr-spinel-absent) layer. Assuming a constant solid fraction in the random loose packing, the volume fraction of plagioclase at the top of this modally graded accumulated layer was ~20 vol.% (i.e. almost half the accumulated volume comprised plagioclase).

#### Crystal aggregation and settling recorded in the PCU

It is clear that olivine was on the liquidus during the solidification of the PCU. Plagioclase was also on the liquidus and grew as suspended crystals in the magma, either from seeds that didn’t settle during the immediate post-emplacement phase, or from seeds entrained from the floor or roof mushy layers by the convecting magma. It will have formed clusters either by synneusis (Vance 1969) or by auto-nucleation (Kirkpatrick 1977) and composite chains and rafts with olivine and/or augite, and have eventually settled on the floor (c.f. Campbell et al. 1978).

The PCU parental liquid lay close to the 5 kbar olivine–plagioclase–augite ternary eutectic (Gibb and Henderson 2006). Although ascent to shallow levels to form the sill would have moved the magma away from augite saturation, only a little cooling would have been required for augite to re-join the liquidus assemblage of the incoming magma.

We suggest that, while the upper picrodolerite was forming, the contemporaneous floor of the PCU comprised a loose framework of settled olivine clusters and plagioclase networks containing interstitial augite. The increasing contribution of plagioclase is likely to have increased the porosity of the crystal accumulation since plagioclase, either as isolated grains or as clusters, forms strongly non-equant particles, and its low density and low settling velocity promote very loose packings (Allen 2001). The porosity of the crystal pile is likely to have been greater than ~45 vol.% and possibly as high as 75 vol.% (e.g. Philpotts et al. 1998, 1999).

Olivine in the crinanite is ophitic, showing that all olivine clusters (with a few rare exceptions) had escaped the convecting magma by the time the top of the accumulating crystal pile had reached ~ 79 m stratigraphic height. However, the presence of rare clusters of olivine in the vicinity of the Sandwich Horizon shows that the magma from which the crinanite crystallised was saturated with olivine, and was likely to contain many small crystals of olivine in addition to the rare large clusters that remained suspended for (almost) the entire solidification history. Following Gibb and Henderson (2006), we suggest that the large ophitic grains of olivine in the crinanite are cored by small crystals that remained suspended, possibly forming relatively buoyant composite clusters with plagioclase that would have remained suspended in the convecting magma for longer than clusters made entirely of olivine.

The original crystal cargo of the incoming magma contained large individual crystals and stellate clusters of plagioclase, and these are distributed through the PCU in a manner consistent with our model of settling from a convecting magma. The relatively low density of plagioclase means that the stellate clusters would remain suspended for longer than olivine aggregates of a similar size. They would then start to settle in appreciable numbers later than the olivine, thus accounting for their stratigraphically higher distribution in the upper part of the picrodolerite. It is surprising that they are not abundant in the upper part of the crinanite, since one might expect them to be easily trapped in the upper solidification front, but this might be an artefact of sampling.

If we assume all olivine above ~33 m stratigraphic height crystallised on an olivine + plagioclase ± augite cotectic and that all the olivine either settled to the floor or became trapped at the roof until the bulk magma became too crystal-rich to permit crystal segregation, the olivine in the upper 46 m of the picrodolerite and in the upper 10 m of the PCU must have crystallised broadly contemporaneously with much of the plagioclase found between 33 m stratigraphic height and the top of the PCU at 158 m. The 11 vol.% of olivine (corrected for overgrowth) in the upper 46 m of the picrodolerite, together with the 10 vol.% in the upper 10 m of the PCU, and the 6 vol.% olivine in the intervening 68 m of crinanite, suggests that the overall olivine mode crystallising from the incoming carrier liquid that formed the PCU, once it had lost its crystal cargo, was ~8 vol.%, consistent with the calculated mode of olivine that crystallised from the liquid PdolPL1 (Gibb and Henderson 2006).

### Microstructural evidence for convection in other sills

The PCU of the Shiant Isles Main Sill is an unusual body, being immediately underlain by a substantial earlier intrusion that was still incompletely solidified (Foland et al. 2000). Cooling was therefore asymmetric, with the greater cooling through the roof providing a driving force for convection. (Although convection may have been dampened by the subsequent intrusion of a 4 m-thick body of olivine-rich picrite above the PCU before the latter had fully solidified, this body is thin compared to the ~23 m of hot rock underlying the PCU). One might therefore argue that the presence of sustained convection in a sill the size of the Shiant Isles Main Sill is unusual and a consequence of its composite nature. However, the literature contains evidence that sills of comparable thickness may also have convected during solidification.

For example, a similar pattern of olivine modal distribution and grain size variation is present in the 220 m-thick sill-like Murotomisaki Gabbroic Complex, with a fine-grained olivine deposit on the sill floor grading upwards into a zone containing many fewer but much larger olivine phenocrysts (Hoshide et al. 2006). This pattern is mirrored at the roof in the same manner as we observe in the Shiant PCU. Hoshide et al. (2006) interpret this as a result of settling of an incoming crystal load, followed by continued growth of the remaining suspended olivine crystals in the upwards-migrating floor solidification front. While no details are provided about grain size or clustering, it is possible that the olivine distribution in the Murotomisaki Gabbroic Complex is a consequence of prolonged settling from a convecting magma.

The plagioclase aspect ratio, quantified as the average apparent aspect ratio viewed in thin section, AR, has been shown to vary systematically across sills, with a strong relationship between AR and model crystallisation times (calculated assuming diffusive heat loss; Holness 2014). At first sight, this demonstrates that plagioclase in the sills examined by Holness (2014) grew in situ in inwards-propagating solidification fronts, rather than as a suspended crystal load in a vigorously convecting magma. However, the symmetrical variation of AR observed in sills thinner than ~40 m is not apparent in either the 130 m-thick Portal Peak sill, nor in the 189 m-thick Koffiefontein sill, in which the minimum value of AR is displaced upwards from the mid-point of the sill (Holness 2014). This is consistent with the accumulation on the floor of relatively non-equant plagioclase grown at the sill roof, suggestive of convective redistribution of material. This will be explored in a later contribution.

## Conclusions

Our microstructural observations are consistent with the Shiant Isles Main Sill being a composite body, as argued by Gibb and Henderson (2014). The early, 24 m thick, picrite sill formed by the intrusion of a highly olivine-phyric magma in which the olivine crystal cargo was concentrated near the centre of the flow. The high particle concentration prevented effective settling of the olivine crystals, which retain their immediately post-emplacement distribution. In contrast, the olivine distribution and microstructures preserved in the next batch of magma to arrive, that which formed the PCU, supports an early period during which much of the crystal load settled to the floor to form a modally and size-sorted accumulation dominated by olivine, followed by a period during which settling of crystals grown in the bulk magma was significantly slowed by convection.

The crystal pile that accumulated during the later period contains olivine grains grown from small remnants of the original crystal cargo that remained suspended following the initial settling phase. The olivine component of the magma crystallised by overgrowth of these grains, which aggregated by synneusis to form clusters that became larger and increasingly better sintered the longer they remained in suspension. The remainder of the accumulated crystal pile on the sill floor was dominated by plagioclase, which was probably also grown predominantly in suspension. Augite grew from the interstitial liquid in the accumulated crystal pile. Once (almost) all large olivine clusters had escaped the convection (at which point the height of the remaining bulk magma was ~70 m) crystal accumulation on the floor was dominated by plagioclase, most likely forming clusters and aggregates with augite and olivine, both of which form large poikilitic grains in the crinanite. The strong asymmetry of the PCU, in which the last horizon to solidify occurs considerably above the mid-point of the intrusion, suggests that significant crystal accumulation occurred on the floor throughout the solidification history.

The combination of a stratigraphically gradual reduction in the mass of settled olivines, a coarsening-upwards of the settled olivines, an upwards increase in the extent of clustering of olivine and the extent to which the clusters are sintered, and the presence of large clusters and grains at the roof strongly support crystallisation during convection in the picrodolerite/crinanite unit of the Shiant Isles Main Sill. A preliminary analysis suggests that the spatial variation of grain size in a polydisperse settled accumulation can also be used to distinguish between settling from static or convecting magma. These results point the way towards a resolution of the question of whether crystallisation in any igneous body was dominated by growth of grains suspended in a convecting magma instead of occurring predominantly in marginal solidification fronts.

## Figures and Tables

**Fig. 13:**
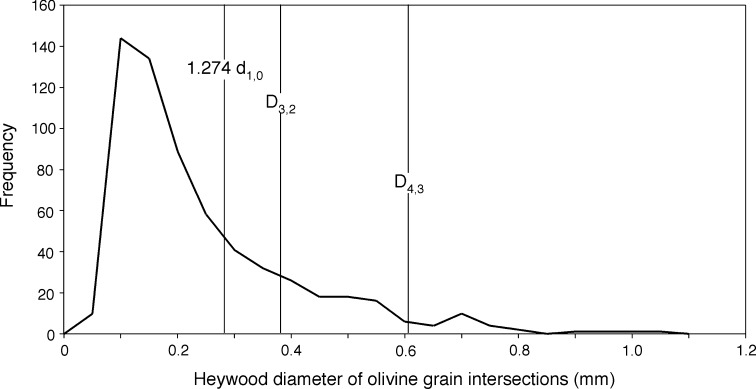
The frequency distribution of Heywood diameters measured for sample SC492 from the Lower Picrite (26.4 m stratigraphic height). Number of measurements is 617. The mean Heywood diameter (d_1,0_) is shown, multiplied by the factor 1.274—this measure provides a good indication of the true 3D diameter of a population of monodisperse spheres. For comparison the area-weighted mean diameter (D_3,2_) and the volume-weighted mean diameter (D_4,3_) are also shown. Data in Table 1

## Appendix

For polydisperse populations, there are numerous types of moment-based (3 dimensional) average diameter, D_p,q_, (Farr et al. 2016), where, for *p* ≠ *q*:

$${{D}_{p,q}}={{\left( \frac{\sum\nolimits_{\text{grains}}{{{D}^{p}}}}{\sum\nolimits_{\text{grains}}{{{D}^{q}}}} \right)}^{1/(p-q)}}.$$

The volume-weighted mean diameter, *D*_4,3_, is dominated by the larger spheres in the distribution and is comparatively insensitive to the tail of fine particles. *D*_4,3_ represents the size around which most of the mass of the particles lies. The area-weighted mean diameter, *D*_3,2_, is the mean that results when spheres are chosen with a probability proportional to their surface area. The area-weighted mean diameter is also less sensitive to the tail of fine particles compared to the simple number-weighted average, and is most appropriate for problems involving surface area, such as the extent and rate of adsorption.

The various different means of the 3D particle size distribution can be determined using thin section observations of populations of grain intersection diameters, *d*, using:

$${{D}_{2,3}}=\left( \frac{3\pi }{8} \right){{d}_{2,1}}$$

$${{D}_{4,3}}=\left( \frac{32}{9\pi } \right){{d}_{3,2}}$$

where:

$${{D}_{i,j}}={{\left( \frac{\Sigma {{d}^{i}}}{\Sigma {{d}^{j}}} \right)}^{1/(i-j)}}$$

A simple measure of the spread of the grain size population, σ, is given by:

$$\sigma =\sqrt{In\left( {{D}_{4,3}}/{{D}_{3,2}} \right)}=\sqrt{In\left( 0.961{{d}_{3,2}}/{{d}_{2,1}} \right)}$$

and an estimate of the uncertainty on the mean diameter, *e*, is provided by:

$$e=\frac{0.15}{\sqrt{n}}\exp \left( 5\sigma \right),$$

where *n* is the number of grain intersections measured (Farr et al. 2016). A comparison of the volume-weighted and area-weighted mean diameters with the commonly used mean Heywood diameter is shown in Fig. 13.

The original grain size of the olivine grains can be determined if we correct the sizes observed in the fully solidified rock to account for any overgrowth of the original phenocrysts during solidification of the carrier liquid. Following Farr et al. (2016), the change in average diameter, δ*D*_i,j_ is given by:

$$\delta {{D}_{i,j}}={{D}_{i,j}}\left[ \frac{\left( i/{{D}_{i,i-1}} \right)-\left( j/{{D}_{j,j-1}} \right)}{i-j} \right]\frac{{{D}_{3,2}}\delta \phi }{3{{\phi }_{\text{sed}}}},$$

where ϕ_sed_ is the initial volume fraction of the olivine and δϕ is the change in volume fraction due to overgrowth
